# Belowground microbiota analysis indicates that *Fusarium* spp. exacerbate grapevine trunk disease

**DOI:** 10.1186/s40793-023-00490-0

**Published:** 2023-04-03

**Authors:** Yonghua Li, Xinghong Li, Wei Zhang, Jiao Zhang, Hui Wang, Junbo Peng, Xuncheng Wang, Jiye Yan

**Affiliations:** 1 Beijing Key Laboratory of Environment Friendly Management on Fruit Diseases and Pests in North China, Institute of Plant Protection, Beijing Academy of Agriculture and Forestry Sciences, Beijing, 100097 China; 2 College of Plant Protection, Hebei Agricultural University, Baoding, 071000 China

**Keywords:** Grapevine trunk diseases, Microbiota, Belowground, *Fusarium* spp., Fungi

## Abstract

**Background:**

Grapevine trunk diseases (GTDs) are disease complexes that are major threats to viticulture in most grapevine growing regions. The microbiomes colonizing plant belowground components form complex associations with plants, play important roles in promoting plant productivity and health in natural environments, and may be related to GTD development. To investigate associations between belowground fungal communities and GTD symptomatic or asymptomatic grapevines, fungal communities associated with three soil–plant compartments (bulk soils, rhizospheres, and roots) were characterized by ITS high-throughput amplicon sequencing across two years.

**Results:**

The fungal community diversity and composition differs according to the soil–plant compartment type (PERMANOVA, *p* < 0.001, 12.04% of variation explained) and sampling year (PERMANOVA, *p* < 0.001, 8.83%), whereas GTD symptomatology exhibited a weaker, but still significant association (PERMANOVA, *p* < 0.001, 1.29%). The effects of the latter were particularly prominent in root and rhizosphere community comparisons. Many GTD-associated pathogens were detected, but their relative abundances were not correlated (or were negatively correlated) to symptomatology. *Fusarium* spp., were enriched in symptomatic roots and rhizospheres compared to asymptomatic counterparts, suggesting that their abundances were positively correlated with symptomatic vines. Inoculation tests revealed that *Fusarium* isolates, similar to *Dactylonectria macrodidyma*, a pathogen associated with black foot disease, caused dark brown necrotic spots on stems in addition to root rot, which blackened lateral roots. Disease indices were higher with co-inoculation than single inoculation with a *Fusarium* isolate or *D. macrodidyma*, suggesting that *Fusarium* spp. can exacerbate disease severity when inoculated with other known GTD-associated pathogens.

**Conclusions:**

The belowground fungal microbiota of grapevines varied from soil–plant compartments, the years and whether showed GTD symptoms. The GTDs symptoms were related to the enrichment of *Fusarium* spp. rather than the relative abundances of GTD pathogens. These results demonstrate the effects of fungal microbiota of roots and rhizospheres on GTDs, while providing new insights into opportunistic pathogenesis of GTDs and potential control practices.

**Supplementary Information:**

The online version contains supplementary material available at 10.1186/s40793-023-00490-0.

## Introduction

Grapevine trunk diseases (GTDs) are among the greatest global challenges in viticulture. GTDs are disease complexes caused by pathogenic wood fungi belonging to approximately 174 species and comprising 32 genera, including *Phaeoacremonium*, *Botryosphaeria*, *Eutypa*, *Diaporthe*, and *Dactylonectria* [1]. Important GTDs include the Esca disease complex (ESCA) [2], Botryosphaeria dieback [3], Eutypa dieback [4], Diaporthe dieback [5], and black foot disease [6]. GTD pathogens often colonize the wood tissues of perennial organs as endophytes, and then transition to pathogens that cause wood necrosis, wood discoloration, vascular infections, and white decay under appropriate conditions [7]. Thus, these infections can spread unnoticed in fields. An effective mechanism to control GTDs is still lacking due to pathogen and pathogenesis complexity. Importantly, because plant replacements are widely used to combat GTDs, GTDs drastically reduce the lifespans of vineyards and increase economic losses. The annual global financial losses due to the replacement of trees killed by GTDs amount to over 1.5 billion dollars [8]. Hence, there is an urgent need for alternative, effective, and environmentally safe strategies to prevent and control GTDs.

GTD development is the result of complex interaction networks within microbial communities and between microorganism populations, GTD-associated pathogens, and hosts. Microbiome interference can lead to widespread changes in hosts and environments [9]. Consequently, strategies of engineering grapevine-associated microbiomes to improve host immunity and/or reduce pathogen virulence represent some of the most promising methods to prevent or control GTDs. Advances in sequencing and meta-omics tools have led to the gradual widespread use of high-throughput sequencing approaches to characterize the microbial communities inhabiting grapevines and gain insights into the factors that contribute to GTD development from an ecological perspective. Because GTDs primarily occur in perennial woods, greater attention has been paid to the bacterial and fungal communities inhabiting aboveground wood associated with symptomatic and asymptomatic plants [10]. Several potential antagonist microorganisms against GTDs have been found to be abundant in asymptomatic vines compared to symptomatic vines, including *Pseudomonas, Bacillus,* and *Streptomyces* [11, 12]*.* Furthermore, diverse GTD pathogens have been detected in both asymptomatic and symptomatic grapevines using culture-independent approaches, suggesting high GTD complexity [13–16]. However, several studies have shown that more severe necrosis is associated with lower relative abundances of GTD pathogens, suggesting that other, yet-to-be identified factors may be involved in GTD development [10, 13, 16]. Thus, additional research is needed to generate insights into the microbial populations associated with GTDs.

Soil microbial communities represent the greatest reservoir of known biological diversity globally, and play crucial roles affecting plant health by influencing plant growth [17, 18], nutrient acquisition [19], and disease resistance [20]. Moreover, they have a largely unexplored potential to expand the genomic capabilities of plants to help meet increasing global demands for crops that are more resilient to stresses (including drought, diseases, and pests) and reduce dependence on fertilizers and pesticides. Soil microbiomes can influence grapevine-associated microbiota and may serve as a bacterial reservoir for aboveground plant components [21]. Grapevine root and rhizosphere microbiomes are also promising potential sources of more efficient and effective GTD biocontrol agents [10]. For example, *Pythium oligandrum* [22–24], *Streptomyces* spp. [24, 25], *Bacillus subtilis* PTA-271, and *Trichoderma atroviride* SC1 [26] have demonstrated inhibitory effects on GTD development. Beneficial colonizing fungi in the root systems of grapevines can reduce ESCA necrosis by enhancing host immunity [22]. These observations highlight the important roles of belowground microbiota in the systemic health of plants and further suggest that belowground microbiota are associated with GTDs. Furthermore, a recent study revealed that GTD pathogens were more abundant in the bulk soils of symptomatic plants [27], but a comprehensive understanding of belowground microbiomes remains lacking, especially regarding the relationships between root, rhizoplane, and rhizosphere microbiomes and GTDs.

To explore whether the belowground fungal communities of grapevines are associated with GTD symptoms, GTD-asymptomatic and symptomatic grapevine samples from three soil–plant compartments (bulk soils, rhizospheres, and roots) were collected in two consecutive years and subjected to ITS amplicon sequencing. The variation and enrichment of fungal populations were analyzed, and enriched fungi were further isolated to evaluate their effects on GTD development. These experiments were used to conduct interannual comparisons of the fungal community composition associated with three soil–plant compartments (bulk soils, rhizospheres, and roots) of symptomatic and asymptomatic vines in the same vineyard for the first time.

## Materials and methods

### Sample collection

Samples were collected from young vineyards in Yinchuan City in the Ningxia Province of China (38.63° N, 106.03° E) in autumn of 2019 and 2020. The samples were collected at similar environmental conditions. It was both cloudy with temperatures ranging from 6–23 ℃ and 8–22 ℃ in 2019 and 2020, respectively. The detailed environmental temperature conditions during the seasons of 2019 and 2020 were listed in Additional file 1: Table S1. The grapevines in the vineyard were a Chardonnay cultivar planted in 2014. This cultivar was reported less susceptible genotypes against the GTD pathogens of *Neofusicoccum parvum* and *Diplodia seriata* [28]. The vineyard soils were alkaline and contained large stones from the Gobi Desert. The physical and chemical characteristics of the soil in the sampling vineyard were listed in Additional file 1: Table S2. No other crops were planted in the area prior to planting grapevines. There were some annual weeds, such as *Convolvulus arvensis* L., *Artemisia halodendron* Turcz.ex Bess and *Portulaca oleracea* L. growing naturally between grapevine rows. No herbicide was used during the sampling years. The records of chemical agents and fertilizers used were listed in Additional file 1: Table S3. GTDs occurred after long-term vineyard observation, with symptoms primarily including chlorotic spots or tiger stripes on the leaves in addition to dark brown necrotic tissues in the cross-sections of the branches and stems (Additional file 2: Fig. S1). Ten vines with typical GTD symptoms were selected as symptomatic trees, while adjacent asymptomatic trees were selected as asymptomatic trees for comparison. The cultivation conditions and growth environments of adjacent sample groups were extremely similar, allowing for the mitigation of noise effects due to different soils and unintended anthropogenic effects. The locations of the vines were recorded and the occurrences of GTDs were investigated in 2019 and 2020, with each vine classified as symptomatic or asymptomatic, followed by sample collection. The lateral root collections were conducted on the same plant each year, unless the tree died after sample collections in 2019 and was replaced with a new vine, then an alternative vine adjacent to the dead one will be selected for sampling in 2020.

Three types of samples were collected, including bulk soils, rhizosphere soils, and roots from each grapevine. The adjacent vine spacing of the same row is 1 m, and the adjacent row spacing is 3.2 m. Soils were collected around the main stem of the grapevine with a sterilized spade in a radius of 40–50 cm and at a depth of 20–30 cm in three directions of an equilateral triangle, including inter-row and inter-grapevine locations. The individual samples were then mixed to represent a bulk soil sample. In total of 20 and 22 bulk soil samples were collected in 2019 and 2020, respectively. The lateral roots of the grapevines were collected with a plastic bag and shaken to remove loosely adhered soil. Samples were then quickly transported back to the laboratory with dry ice. Lateral roots were washed in the laboratory with sterilized 1 × PBS until no visible soil particles remained and the PBS solution was clear. Then the resultant suspension was centrifuged at 7000 × *g* for 2 min. The soil obtained after centrifugation was used to represent the rhizosphere soil sample. The remaining root tissues were considered to only contain rhizoplane and endophytic microorganisms. Resultant samples were then divided into two components, with one used for immediate fungal isolation and the other stored at − 80 °C for subsequent DNA extraction and sequencing. A total of 297 samples were used in this study for molecular analysis, including 127 root samples, 128 rhizosphere soil samples and 42 bulk soil samples.

### DNA extraction, PCR amplification, and sequencing

Soil and root samples were subjected to fungal ITS community profiling via sequencing. Soil and root sample DNA were extracted using a FastDNA SPIN Kit (MP Biomedicals, California, USA) and the CTAB method, respectively. The DNA concentration and purity were evaluated with 1% agarose gel electrophoresis. ITS1 regions were amplified using the primer set ITS-1F-F (5′-3′: CTTGGTCATTTAGAGGAAGTAA) and ITS1-1F-R (5′-3′: GCTGCGTTCTTCATCGATGC) [29]. PCR reactions were conducted with Phusion® High-Fidelity PCR Master Mix (New England Biolabs, Ipswich, Massachusetts, USA), 0.2 µM each of forward and reverse primers, and about 10 ng of template DNA. PCRs were conducted with an initial denaturation step at 98 °C for 1 min to activate the polymerase, followed by amplification with 30 cycles of denaturation at 98 °C for 10 s, annealing at 50 °C for 30 s, elongation at 72 °C for 30 s, and a final extension at 72 °C for 5 min. PCR products were detected using 2% agarose gel electrophoresis and individually quantified using a Qubit 2.0 fluorometer (Thermo Fisher Scientific, Massachusetts, USA), followed by mixing in equimolar concentrations. The resultant PCR mixture pool was purified with a Qiagen Gel Extraction Kit (Qiagen, Dusseldorf, Germany). Sequencing libraries were then generated using the TruSeq® DNA PCR-Free Sample Preparation Kit (Illumina, San Diego, California, USA), following the manufacturer's protocols to attach indexing oligonucleotides. The library quality was assessed using a Qubit 2.0 Fluorometer and the Agilent Bioanalyzer 2100 system (Palo Alto, California, USA). Finally, the library was sequenced on the Illumina NovaSeq platform (Novogene, Beijing, China) to generate 250 bp paired-end reads.

### Data analysis

Raw reads from each sample were assembled into longer DNA tags using the FLASH program (v.1.2.7, http://ccb.jhu.edu/software/FLASH/) [30]. The resultant sequences were quality-filtered using strictly-defined processes [31] using the split_libraries_fastq.py script of QIIME (v.1.9.1) [32]. Chimeric sequences were identified and removed using the VSEARCH software program [33]. The resultant clean sequences were clustered at a 97% nucleotide sequence identity as operational taxonomic units (OTUs) using the UPARSE tool (v.7.0.1001) [34]. Sequences with the highest frequency in read counts were designated as the representative sequences for each OTU. Each representative sequence was annotated using the assign_taxonomy.py script of QIIME and comparison against the UNITE Database [35, 36] (v.7.2, https://unite.ut.ee/) using the BLAST method. To investigate the phylogenetic relationships among different OTUs, a multiple sequence alignment was generated for dominant sequences using the MUSCLE software program (v.3.8.31, http://www.drive5.com/muscle/) [37].

To compare OTU compositions among samples, OTU abundance information was normalized using a standard sequence number corresponding to the sample with the least amount of sequences. Subsequent alpha and beta diversity analyses were performed using the normalized data. Alpha diversity, which is used to analyze species diversity, was estimated using five indices, including the observed-species, Chao1, Shannon, Simpson, and ACE metrics, which were calculated with QIIME and then visualized in R. Multiple mean comparisons were performed using the Wilcoxon test to determine how fungal community alpha diversity differed among sample groups. Relationships of OTU composition among samples were investigated by calculating beta diversity metrics in QIIME. Furthermore, principal coordinates analysis (PCoA) was conducted to visualize the variation in community composition using the vegan and ggplot2 packages in R software. PERMANOVA tests were also used to investigate the OTUs that significantly differed in abundance among experimental factors using the vegan package, wherein the test R^2^ values indicated the relative variable importance. In addition, linear discriminant analysis effect size (LEfSe) tests [38] were used to identify taxa that differed in relative abundance among sample groups and that could be considered biomarkers for sample groups. The threshold logarithmic linear discriminant analysis (LDA) score for identifying distinguishing taxa was set at 3.0, and the Wilcoxon *p* value was set at 0.05.

### Isolation, cultivation, and identification of *Fusarium* spp.

For fungal isolation, root tissues were cut into small tissue blocks of about 0.5 cm in length and then surface-disinfected by applying 75% ethanol for 30 s and 2% NaClO for 5 min. After being washed five to seven times with sterilized water, the tissue blocks were dried on sterilized filter paper on a clean bench. The root blocks were then placed on a potato dextrose agar (PDA) plate (four blocks per plate), sealed, and cultured at 27 °C in alternating dark (13 h) and light (11 h) conditions. To isolate fungi from rhizosphere and bulk soils, the soil concentration gradient dilution method was used. Specifically, 1 g of soil was added to 9 mL of 1 × PBS solution in a 15-mL Eppendorf tube, shaken, and mixed to generate a soil suspension to obtain a concentration of 10^−1^. Additional dilution of the soil suspension was conducted to obtain soil diluents at concentrations of 10^−2^–10^−5^. Samples from each soil diluent were then used as inocula and evenly spread on PDA plates. To obtain single spore cultures, conidia were diluted with sterilized ddH_2_O and spread on 1.5% water agar (WA) plates, followed by incubation at 27 °C until germination. A single germinating spore was then transferred to a new PDA plate and incubated at 27 °C for 5–7 days. Colony diameters, in addition to the shapes, sizes, and color of conidia, were observed using Axiocam 506 color Imager Z2 photographic microscope (Carl Zeiss Microscopy GmbH, Jena, Germany) and recorded using the ZEN Pro 2012 software program (Carl Zeiss Microscopy). The pure cultures obtained in this study were deposited in the culture collection of the Institute of Plant and Environmental Protection at the Beijing Academy of Agriculture and Forestry Sciences (JZB) in Beijing, China. The strain numbers are JZB3110089, JZB3110090, JZB3110102, JZB3110103 and JZB3110180. The colony and spore morphologies of all obtained fungi were observed, with sickle-shaped types selected as candidate *Fusarium* spp. strains. A total of 50–100 mg mycelia collected from candidate strains was cultured for seven days and total DNA was extracted using the CTAB method. The ITS sequences of the candidate strains were then amplified to identify strains at the genus level using the primers ITS1 (5′-3′: TCCGTAGGTGAACCTGCGG) and ITS4 (5′-3′: TCCTCCGCTTATTGATATGC) (White et al., 1990), followed by amplicon sequencing. The resultant DNA sequences were compared against the NCBI NT database using the BLASTn tool (https://blast.ncbi.nlm.nih.gov/Blast.cgi). Five strains encoding ITS sequences assigned as *Fusarium* and with spores that were sickle-shaped were retained for further analysis. The five strains were used as representative isolates for pathogenicity tests.

### Pathogenicity tests

Young, healthy, 2-year-old rooted Chardonnay cuttings in pots were used to assess the pathogenicity of the representative isolates JZB3110089, JZB3110090, JZB3110103, JZB3110102, and JZB3110180 in addition to the black foot disease-associated fungi *Dactylonectria macrodidyma* JZB3310008, which was previously isolated from the vineyard. Isolates were cultured on sterilized wheat grains in 500-mL conical bottles until mycelia covered all of the wheat grains. The roots and stem bases were cleaned in running tap water, then surface-sterilized in 70% ethanol for 5 s, followed by three final rinses with sterilized water. Then, 1–2-cm sections were cut from the tips of the sterilized roots to create wounds. A mixture of nutrient soil and vermiculite was established as the cultivation substrate at a ratio of 1:1 (v:v) and sterilized twice at 121 °C for 20 min. The mycelium-covered wheat grains and sterilized cultivation substrate were mixed at a ratio of 1:100 (v:v), and distributed into sterilized pots. The potted grape seedlings were transferred to a greenhouse and cultivated at a temperature of 28 °C and a humidity level of 60%. The seedlings were separately inoculated with the GTD causal agents and *Fusarium* isolates and then co-inoculated at a ratio of 1:1 (v:v). Control plants were planted in sterilized cultivation substrates with sterilized wheat grains. The experiment was replicated for each condition using four plants. Disease symptoms were regularly observed and phenotypic statistics were analyzed seven months after inoculation. Plants were rated on a 0–4 scale according to the ratio of the diseased lateral roots indicated by the blackened transverse section, where 0 = 0%, 1 = 1–25%, 2 = 26–50%, 3 = 51–75%, and 4 = 76–100%. The severity of disease was indicated by the disease index, which was calculated using the following formula:

$$\begin{aligned} {\text{Percent}}\;{\text{disease}}\;{\text{index}} & = {\text{sum}}\;{\text{of}}\;{\text{numerical}}\;{\text{ratings}}/\left( {{\text{number}}\;{\text{of}}\;{\text{plants}}\;{\text{examined}}} \right. \\ & \quad \left. { \times {\text{maximum}}\;{\text{grade}}} \right) \times {1}00\% . \\ \end{aligned}$$

Strains were then re-isolated from root tissues to confirm their identity via Koch's postulates.

## Results

### High-throughput amplicon sequencing

A total of 19,192,952 fungal ITS reads were obtained from 297 samples after paired-end alignments, quality filtering, and the removal of chimeric sequences. The sequences were assigned to 5251 fungal OTUs, 941 of which were classified at the genus level, along with 1529 classified at the species level, both comprising 76.3% of all reads (Table 1). The numbers of reads for roots, rhizosphere and bulk soils were 64,825.53 ± 3803.05, 64,589.54 ± 3331.31 and 64,110.69 ± 2983.33, respectively (Additional file 1: Table S4). Alpha diversity index values for the Shannon, Simpson, Chao1, and ACE metrics ranged from 0.609–6.387, 0.122–0.971, 107.67–993, and 113.208–1,015.505, respectively (Additional file 1: Table S5). The species accumulation curves were close to saturation with increasing sequencing numbers, indicating that the sequencing depth used was sufficient to cover actual diversity within the samples (Additional file 2: Fig. S2). Sequencing data were deposited under the Bioproject accession number PRJNA831299.

**Table 1:** Composition of fungal taxa identified among all samples analyzed here

	Kingdom	Phylum	Class	Order	Family	Genus	Species
Ratio (%)	100	99.41	76.39	76.33	76.33	76.33	76.33
Classified OTUs	5251	4884	3783	3744	3744	3744	3744
Identified taxa	1	17	67	167	401	941	1529
Number of reads	19,192,952	19,079,051	14,662,033	14,650,062	14,650,062	14,650,062	14,650,062

### Sampling year affected grapevine belowground fungal community composition

Samples were collected from different soil–plant compartments, including the roots, rhizosphere, and bulk soil, in order to explore the correlation between soil–plant compartments and grapevine fungal communities. Although rhizosphere soils were removed by cleaning the roots using PBS, microorganisms on the root surfaces would not have been entirely removed. Indeed, the sequencing of root samples revealed the presence of fungi on the root surfaces and in the roots. Consequently, these samples were referred to as PE samples for simplicity, corresponding to rhizo**p**lane and **e**ndophyte samples. **R**hizosphere and **b**ulk soils were accordingly referred to as R and B samples, respectively, while samples collected in 2019 and 2020 were delineated by 19 and 20, respectively. In addition, samples collected from **as**ymptomatic and **s**ymptomatic trees were referred as As and S, respectively. For example, sample AsPE.19 represented the rhizoplane and endophyte fungi for samples collected from asymptomatic grapevines in 2019.

**Fig. 1:**
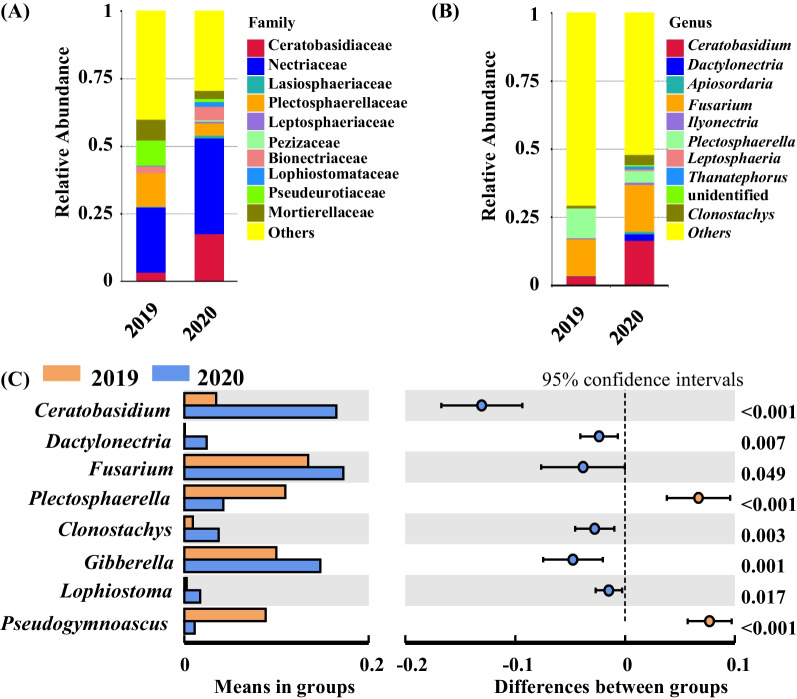
Differences in relative abundances of fungi in the belowground components of grapevines between 2019 and 2020. **A** and **B** The relative abundances of the top 10 most abundant fungal taxa at the family and genus levels, respectively. The less abundant taxa are referred to as “others”. **C** shows genera with significant differences in relative abundance between 2019 and 2020. *t* test, *p* < 0.05

The relative abundances of dominant fungal taxa changed significantly between 2019 and 2020. In 2019, the most abundant families were Nectriaceae, Plectosphaerellaceae, and Pseudeurotiaceae, accounting for 24%, 12.52%, and 9.33% relative abundances of all sequence datasets, respectively (Fig. 1A). In addition, the most abundant genera were *Fusarium* (13.42%), *Plectosphaerella* (10.95%), and *Gibberella* (10%) (Fig. 1B). However, the most abundant families in 2020 were Nectriaceae, Ceratobasidiaceae, and Bionectriaceae, accounting for 35.49%, 17.64%, and 4.50% of all sequence datasets, respectively (Fig. 1A). In addition, the most abundant genera in 2020 were *Fusarium* (17.24%), *Ceratobasidium* (16.50%), and *Gibberella* (14.75%) (Fig. 1B). Among genera with relative abundance greater than 1%, *Ceratobasidium*, *Dactylonectria*, *Fusarium*, *Clonostachys*, *Gibberella*, and *Lophiostoma* exhibited significantly higher relative abundances in 2020 samples compared to 2019 samples, while the relative abundances of *Plectosphaerella* and *Pseudogymnoascus* significantly decreased (*t* test, *p* < 0.05, Fig. 1C).

**Fig. 2:**
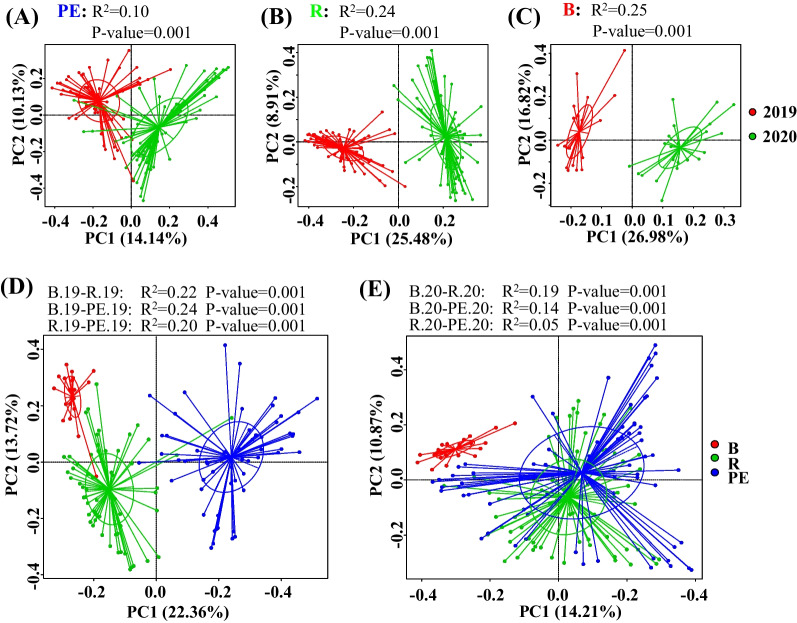
Principal coordinate analysis (PCoA) based on Bray–Curtis dissimilarity metrics. **A**–**C** The distances in the fungal communities of roots, rhizospheres, and bulk soils, respectively, between the sampling years of 2019 and 2020. The distances of different soil–plant compartments in fungal communities collected in 2019 and 2020 are shown in (**D**) and (**E**), respectively. The PERMANOVA test was used for statistical analysis

Significant differences in alpha-diversity index values were observed between the pairs PE.19–PE.20, R.19–R.20, and B.19–B.20 (Additional file 2: Fig. S3). Specifically, samples collected in 2020 exhibited significantly lower Shannon, Simpson, Chao1, and ACE values than those collected in 2019 (Wilcoxon test, 0.01 < *p* < 0.05 for the Simpson index of the B.19–B.20 pair, and *p* < 0.001 for the other comparison pairs). In addition, PERMANOVA analysis revealed that the sampling year was significantly associated with total fungal community variation in grapevine belowground components, contributing to 8.83% of total variation (*p* < 0.001). Significant differences (*p* = 0.001) were also observed between the PE.19–PE.20, R.19–R.20, and B.19–B.20 pairs, with R^2^ values of 0.10, 0.24, and 0.25 respectively (Fig. 2ABC). The compositional variation in fungal microbiota between these pairs was analyzed with PCoA based on the Bray–Curtis dissimilarity metric. Samples collected in 2019 and 2020 clustered separately in the analysis (Fig. 2ABC). In addition to the above analyses, the level of OTU exclusivity among samples was visualized using Venn diagrams (Additional file 2: Fig. S4). In total, 1263 OTUs were exclusive to PE.19 compared to PE.20, while 963 OTUs were exclusive to PE.20 and 903 OTUs were shared by the two (Additional file 2: Fig. S4A). In addition, 1231 and 1146 OTUs were specific to R.19 and R.20, respectively, with 903 OTUs shared between them (Additional file 2: Fig. S4B). Lastly, 857 and 436 OTUs were specific to B.19 and B.20, respectively, with 953 OTUs shared between them (Additional file 2: Fig. S4C). Overall, these results indicated that sampling time was significantly associated with fungal community composition, with the 2019 samples exhibiting greater diversity in fungal communities compared to 2020 communities.

### Fungal community composition differed among soil–plant compartments

In total, 1866, 2550, and 1828 OTUs were observed in the PE.19, R.19, and B.19 samples, respectively. Among these, 577, 605, and 207 OTUs were exclusive to PE.19, R.19, and B.19, respectively, comprising 30.92%, 23.72%, and 11.32% of their total OTUs, respectively (Additional file 2: Fig. S4C). Further alpha diversity analysis revealed that B.19 exhibited the highest Shannon, Simpson, Chao1, and ACE values, followed by R.19 and PE.19 (Additional file 2: Fig. S3). In addition, significant differences were observed for the Shannon, Chao1, and ACE indices between the pairs of PE.19–R.19, PE.19–B.19, and R.19–B.19 (Additional file 2: Fig. S3ACD). Significant differences were also observed for the Simpson index between these group pairs, except for the R.19–B.19 pair (Wilcoxon test, *p* < 0.001, Additional file 2: Fig. S3B). Moreover, PCoA based on Bray–Curtis compositional dissimilarities revealed significant clustering of fungal communities based on soil–plant compartments (PERMANOVA, *p* = 0.001) (Fig. 2D). The R^2^ values for these comparisons were 0.2, 0.24, and 0.22 for R.19–PE.19, B.19–PE.19, and B.19–R.19, respectively.

Similar results were obtained for the 2020 communities, with 2166, 2465, and 1389 OTUs observed in the PE.20, R.20, and B.20 communities, respectively. Among these, 878, 909, and 395 OTUs were specific to the PE.20, R.20, and B.20 samples, accounting for 40.58%, 36.88%, and 28.44% of their total OTUs, respectively (Additional file 2: Fig. S4E). Significant differences and similar trends were observed for the Shannon, Simpson, Chao1, and ACE values when comparing the PE.20–R.20, PE.20–B.20, and R.20–B.20 pairs (Additional file 2: Fig. S3). Biological replicates for each soil–plant compartment tended to cluster together and were significantly separated from those from other compartments (PERMANOVA, *p* = 0.001) in the PCoA ordinations (Fig. 2E). Between-pair R^2^ values were 0.05, 0.14, and 0.19 for PE.20–R.20, PE.20–B.20, and R.20–B.20, respectively. PERMANOVA analysis also revealed that soil–plant compartments affected the total fungal community variation in grapevine belowground components, contributing to 12.04% of the overall variation (*p* < 0.001). These results suggested that fungal communities differed significantly among soil–plant compartments, and that bulk soil harbored the highest fungal diversity, followed by rhizosphere soil and roots.

### The influence of symptomatology on grapevine belowground fungal communities

Comparison of fungal communities in symptomatic and asymptomatic plants and in different soil–plant compartments from 2019 and 2020 revealed 248 total OTUs in all communities, reflecting the core grapevine microbiota (Additional file 2: Fig. S5A). The most abundant family was Nectriaceae, comprising 24.19% of the core microbiota, followed by Chaetomiaceae (5.65%) and Hypocreales_fam_Incertae_sedis (4.44%) (Additional file 2: Fig. S5B). *Fusarium* exhibited the highest relative abundance in the overall fungal communities, and was also the most abundant genus in the core microbiota with a relative abundance of 17.74%, followed by *Gibberella* (4.84%) and *Aspergillus* (3.63%) (Additional file 2: Fig. S5C).

**Fig. 3:**
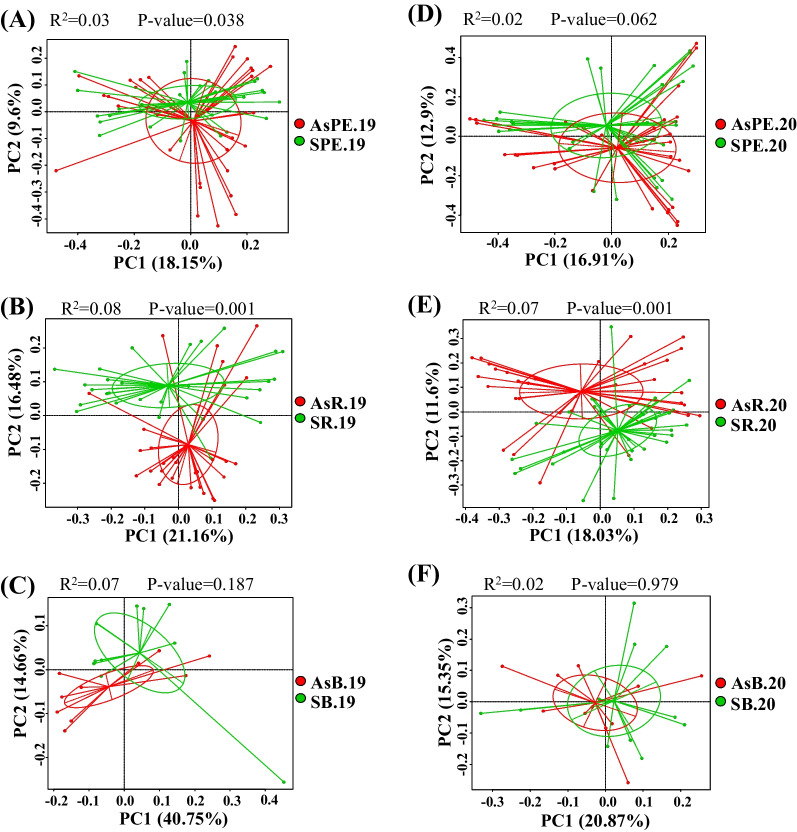
Principal coordinate analysis (PCoA) based on Bray–Curtis dissimilarity metrics showing the distance in the fungal communities of asymptomatic and symptomatic roots collected in 2019 (**A**), asymptomatic and symptomatic rhizospheres collected in 2019 (**B**), asymptomatic and symptomatic bulk soils collected in 2019 (**C**), asymptomatic and symptomatic roots collected in 2020 (**D**), asymptomatic and symptomatic rhizospheres collected in 2020 (**E**), and asymptomatic and symptomatic bulk soils collected in 2020 (**F**). The PERMANOVA test was used for statistical analysis

Significant differences were observed in the Shannon and Simpson index values between the AsPE.19 and SPE.19 pair, highlighting the higher diversity of fungal taxa in the roots of symptomatic grapevines (Wilcoxon test, 0.001 < *p* < 0.01). Root samples collected in 2020 exhibited a similar trend in Shannon and Simpson indices, although these differences were not significant (Additional file 2: Fig. S6AB, Additional file 1: Table S5). GTD symptomatology exhibited a weak, but still statistically significant effect on total fungal community variation in the belowground grapevine components (1.29% total contribution, PERMANOVA, *p* < 0.001). The compositional variation in fungal communities was evaluated using PCoA based on Bray–Curtis dissimilarity values, revealing the clustering of samples by symptomatology. PERMANOVA analysis also revealed significant differences (*p* = 0.038) between asymptomatic and symptomatic root communities collected in 2019 (R^2^ = 0.03), although no significant differences were observed in samples collected in 2020 (Fig. 3AD). Significant differences (*p* = 0.001) were observed between asymptomatic and symptomatic rhizosphere communities collected in 2019 and 2020 (R^2^ = 0.03 and 0.07, respectively) (Fig. 3BE). Significant differences were not observed between community compositions in asymptomatic and symptomatic bulk soils in 2019, nor in 2020 (Fig. 3CF).

**Fig. 4:**
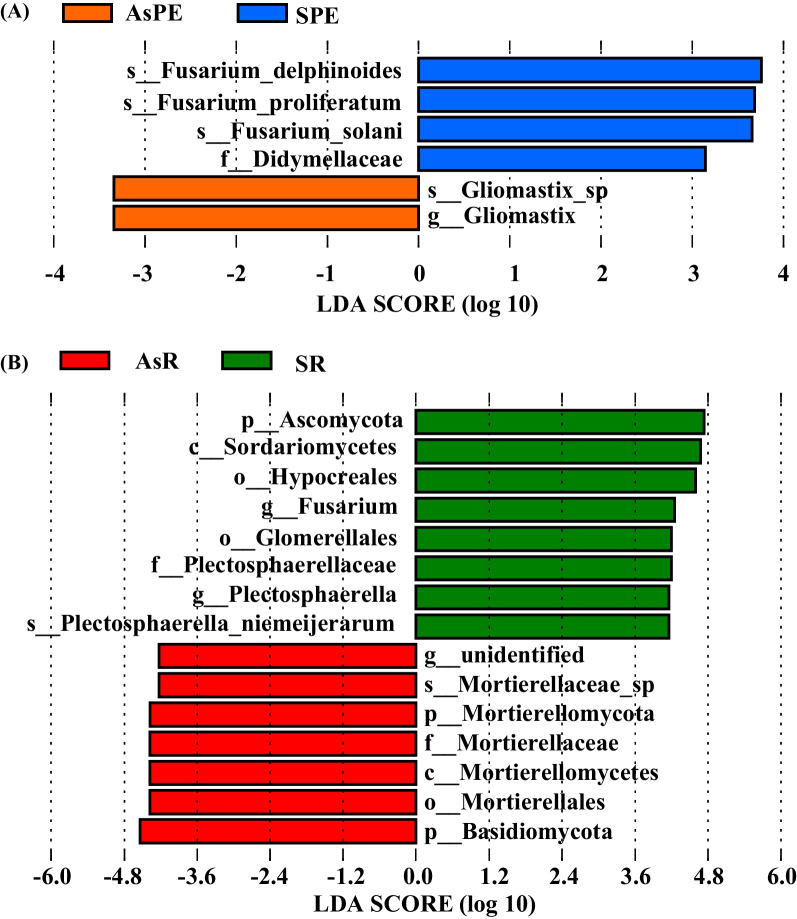
Linear discriminant analysis effect size (LEfSe) analysis of fungal taxa enrichment among treatments. Fungal biomarker enrichment among AsPE (the rhizoplane and endophyte fungi for samples collected from asymptomatic grapevines) and SPE (the rhizoplane and endophyte fungi for samples collected from symptomatic grapevines) with LDA values > 3 are shown in (**A**), and asymptomatic and symptomatic rhizosphere soils with LDA values > 4 are shown in (**B**). p, phylum; c, class; o, order; f, family; g, genus; s, species. LDA, linear discriminant analysis

To identify the taxa that were the most likely to be relevant to symptomatology, the same types of samples collected in 2019 and 2020 were combined and analyzed together. A total of 441 OTUs were shared by AsPE and SPE, with 265 and 132 OTUs exclusive to asymptomatic and symptomatic roots, respectively (Additional file 2: Fig. S7A, Additional file 1: Table S6). *Fusarium* spp., *Mortierella* spp., and *Filobasidium* spp. were the most common fungi in asymptomatic roots, with relative abundances of 3.29%, 1.37%, and 1.37%, respectively. *Fusarium* spp., *Penicillium* spp., and *Westerdykella* spp. were the most common fungi in symptomatic roots, with relative abundances of 7.58%, 3.03%, and 1.52%, respectively. LEfSe analysis identified several fungal taxa that were specifically enriched under different disease states (Fig. 4A). The three species *Fusarium delphinoides*, *Fusarium proliferatum*, and *Fusarium solani*, in addition to the family Didymellaceae, were significantly more abundant in symptomatic roots (LDA scores of 3.75, 3.68, 3.65, and 3.14, respectively) (*p* < 0.01). Because *Fusarium* spp. infect crops globally, the enrichment of *Fusarium* in symptomatic samples could be related to the occurrence of GTD.

Symptomatic rhizosphere sample communities collected in 2019 and 2020 exhibited significantly lower Shannon, Simpson, Chao1, and ACE values in comparison with asymptomatic rhizosphere communities (Additional file 2: Fig. S6) (Wilcoxon test, *p* < 0.05). In addition, asymptomatic and symptomatic rhizosphere group replicates clustered in significantly separated groups in PCoA plots based on the Bray–Curtis dissimilarity metric (PERMANOVA, *p* = 0.001), with R^2^ values of 0.08 and 0.07 for the samples collected in 2019 and 2020, respectively (Fig. 3BE). A total of 680 OTUs were common to the rhizospheres of asymptomatic and symptomatic plants from both the 2019 and 2020 sampling years (Additional file 2: Fig. S7B, Additional file 1: Table S6). These results suggested that fungal diversity was lower in symptomatic plant rhizospheres compared with those of asymptomatic plants, and that the fungal communities of grapevine rhizospheres could be related to GTD occurrence. A total of 336 OTUs were exclusive to the rhizospheres of asymptomatic plants, with 163 identified genera. Dominant genera included *Mortierella* spp., *Fusarium* spp., and *Microascus* spp., which accounted for 2.68%, 2.68%, and 1.19% of these OTUs, respectively. A total of 211 OTUs were specific to the rhizospheres of symptomatic plants, with *Ascomycota* spp., *Fusarium* spp., and *Cladosporium* spp. being the most abundant genera, comprising 3.79%, 2.84%, and 1.90% of these OTUs, respectively. As observed for the AsPE and SPE comparison, *Fusarium* spp. were enriched in the rhizosphere communities of symptomatic plants (LEfSe analysis, LDA = 4.25, *p* < 0.01; Fig. 4B) when compared to those of asymptomatic plants, further suggesting that *Fusarium* enrichment could be related to GTD occurrence. In addition, several taxa assigned at different classification levels exhibited significantly higher relative abundances in the rhizospheres of symptomatic plants than in those of asymptomatic plants, including the phylum Ascomycota, the class Sordariomycetes, the order Hypocreales, the order Glomerellales, the family Plectosphaerellaceae, the genus *Plectosphaerella*, and the species *Plectosphaerella niemeijerarum* (LEfSe analysis, LDA > 4.25, *p* < 0.05, Fig. 4B). The Mortierellaceae family was enriched in rhizosphere soils of asymptomatic plants.

### Distribution of pathogens associated with GTDs

**Fig. 5:**
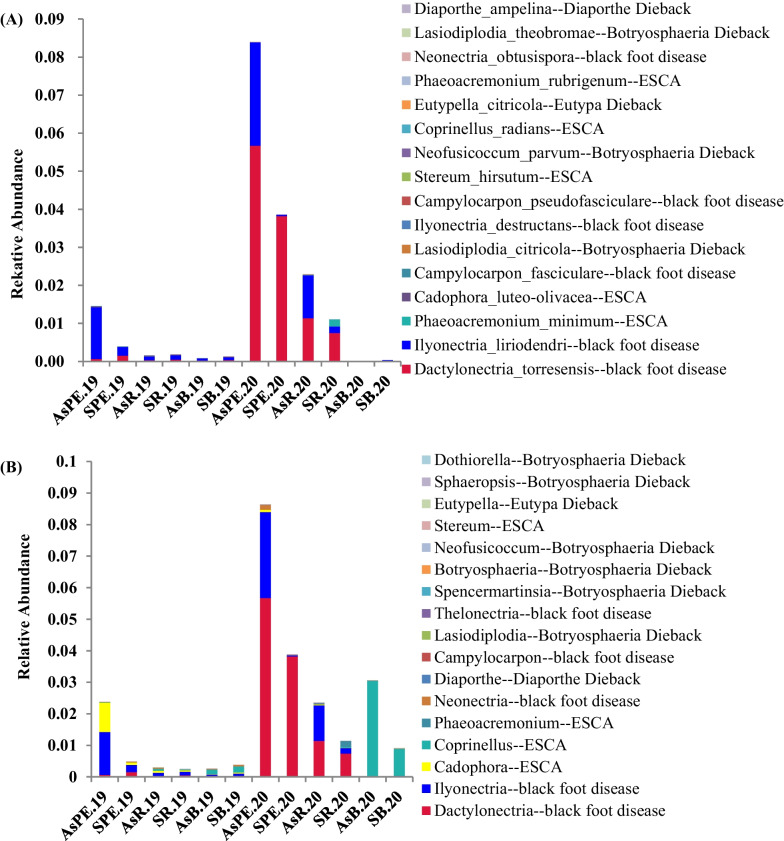
Relative abundances of grapevine trunk disease (GTD)-associated fungi detected in this study based on ITS amplicon sequencing at the species (**A**) and genus (**B**) levels

To identify the distribution of pathogens associated with GTDs in grapevine root samples, 174 species belonging to 32 genera that were previously identified in [1] and that were associated with ESCA, Botryosphaeria dieback, Eutypa dieback, Diaporthe dieback, and black foot disease were examined. A total of 16 species previously reported as pathogens associated with GTDs were detected in the samples collected in this study. In addition, 17 genera that contained GTD-associated pathogens were also detected (Additional file 1: Table S7). Pathogenic *Dactylonectria* spp. that were associated with black foot disease exhibited the highest relative abundances (5.67% in AsPE.20; Fig. 5), consistent with the isolation of causal agents of GTDs in the same vineyard [39]. Pathogens associated with black foot disease included *Dactylonectria torresensis*, *Campylocarpon fasciculare*, *Campylocarpon pseudofasciculare*, *Ilyonectria destructans*, *Ilyonectria liriodendra*, and *Neonectria obtusispora*. Furthermore, pathogens associated with Botryosphaeria dieback were also detected, including *Lasiodiplodia citricola*, *Lasiodiplodia theobromae*, and *Neofusicoccum parvum*. *Diaporthe ampelina* and *Eutypella citricola*, which are associated with Diaporthe dieback and Eutypa dieback, respectively, were also detected. In addition, five species associated with ESCA were detected, including *Cadophora luteo-olivacea*, *Coprinellus radians*, *Phaeoacremonium minimum*, *Phaeocremonium rubrigenum*, and *Stereum hirsutum*.

**Fig. 6:**
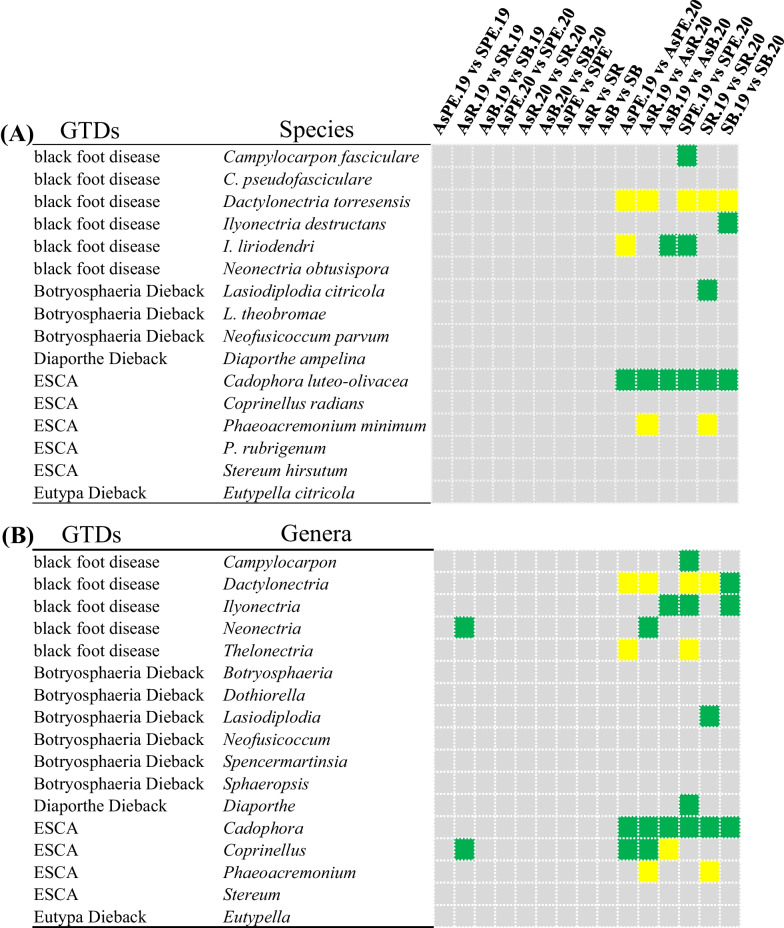
Relative abundance differences of grapevine trunk disease (GTD)-associated fungi at the species (**A**) and genus (**B**) levels between comparable groups are indicated in the upper right side of the figure. Grey indicates no significant difference in the relative abundance of a certain species or genus between the groups being compared, while green and yellow indicate significant differences. Green indicates that the relative abundance of the former group is significantly higher than that of the latter one, while yellow indicates the opposite. For example, when AsPE.19 (the rhizoplane and endophyte fungi for samples collected from asymptomatic grapevines in 2019) was compared with AsPE.20 (the rhizoplane and endophyte fungi for samples collected from asymptomatic grapevines in 2020) (AsPE.19 vs. AsPE.20), the relative abundance of *Dactylonectria torresensis* in the former group was significantly lower than that in the latter group (indicated in yellow). The relative abundance of *Cadophora luteo-olivacea* was significantly higher in the former group than in the latter one (indicated in green). Statistical differences are indicated by q-values < 0.05 based on Wilcoxon tests

The relative abundances of GTD causal agents exhibited significant differences between the 2019 and 2020 sample communities. At the species level (Fig. 6A, Additional file 1: Table S8), the relative abundances of *D. torresensis* in the root and rhizosphere communities collected in 2019 were significantly lower than in the 2020 communities (Wilcoxon test, q < 0.01). In addition, *C. luteo-olivacea*, which is associated with ESCA, was identified in all samples collected in 2020 and exhibited significantly lower relative abundances than in 2019 (Wilcoxon test, q < 0.05). In addition, the pathogens *I. liriodendri* and *L. citricola*, which are associated with black foot disease and Botryosphaeria dieback, respectively, also exhibited significant interannual differences in relative abundances among several groups (Wilcoxon test, q < 0.05). At the genus level (Fig. 6B, Additional file 1: Table S8), *Campylocarpon*, *Dactylonectria*, *Thelonectria*, *Botryosphaeria*, *Lasiodiplodia*, *Diaporthe*, *Cadophora*, *Coprinellus*, and *Phaeoacremonium* exhibited significant interannual differences in relative abundances (Wilcoxon test, q < 0.05).

To investigate the distribution of GTD causal agents in the belowground compartments of grapevines, the relative abundances of these pathogens were compared among roots, rhizosphere, and bulk soil communities. The *Dactylonectria* spp. pathogens, which are associated with black foot disease, exhibited the highest relative abundances in roots, followed by within rhizosphere and bulk soil samples. Similar distribution trends were also observed for *Ilyonectria* spp., *Diaporthe* spp., and *Cadophora* spp., which are associated with black foot disease, Diaporthe dieback, and ESCA, respectively (Additional file 2: Fig. S8). *Lasiodiplodia* spp., which are associated with Botryosphaeria dieback, were enriched in the rhizospheres of asymptomatic samples collected in 2020 (Additional file 2: Fig. S8C). Furthermore, *Diaporthe* spp. and *Campylocarpon* spp., which are associated with Diaporthe dieback and black foot disease, respectively, were enriched in the rhizosphere tissues of symptomatic samples collected in 2020 (Additional file 2: Fig. S8D). Specifically, *Coprinellus* spp., which are associated with ESCA, were enriched in the bulk soils of both asymptomatic and symptomatic samples collected in 2019 and 2020 (Additional file 2: Fig. S8).

To explore whether GTD occurrence was caused by the enrichment of certain pathogens, relative abundances were compared between symptomatic and asymptomatic samples from the same sampling year and within the same soil–plant compartments. At the species level, the relative abundances of the dominant *D. torresensis* pathogenic taxa, in addition to other pathogens, were not significantly different in symptomatic and asymptomatic samples. However, statistically significant differences were observed in two genera in contrast to expectations. The genera *Neonectria* and *Coprinellus* exhibited significantly higher relative abundances in asymptomatic rhizospheres compared to symptomatic rhizospheres collected in 2019 (Wilcoxon test, q < 0.05, Fig. 6B). Thus, these results suggested that differences in disease occurrence between two adjacent vines were not necessarily due to differences in the relative abundances of known GTD pathogens.

The causal agents of GTDs are commonly detected in the aboveground components of grapevines via traditional isolation and identification methods. In this study, diverse pathogens associated with GTDs were detected in the belowground components of grapevines using high-throughput sequencing and culture-independent approaches. The results suggest that GTD symptoms may not occur due to a single GTD, but rather due to a combination of several GTDs. Thus, the causal agents of GTDs in belowground components may promote the occurrence of symptoms in aboveground components. In addition, very few pathogens exhibited significant differences in relative abundances between asymptomatic and symptomatic samples, suggesting that differences in the relative abundances of pathogens were not caused by differences in the symptoms of adjacent grapevines, but rather that the enrichment of *Fusarium* in symptomatic samples might be related to GTD development.

### *Fusarium* spp. grapevine pathogens exacerbate GTDs

**Fig. 7:**
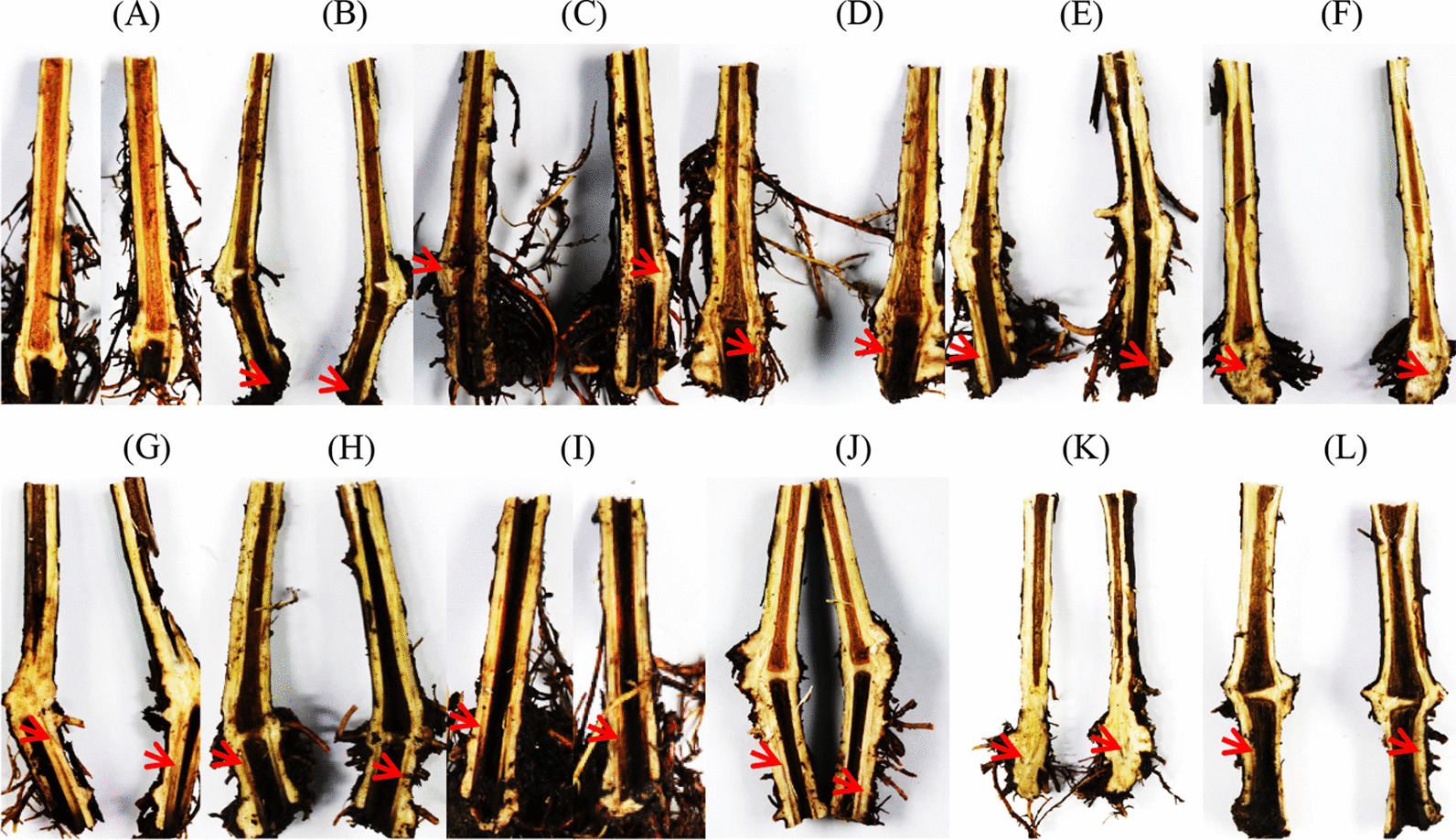
Grapevine stem symptoms after inoculation with *Fusarium* and *Dactylonectria macrodidyma* isolates. **A** Control, **B** *Fusarium* sp. JZB3110090; **C** *Fusarium* sp. JZB3110089; **D** *Fusarium* sp. JZB3110102; **E** *Fusarium* sp. JZB3110103; **F** *Fusarium* sp. JZB3110180; **G** *D. macrodidyma* JZB3310008 associated with Black Foot Disease; **H** *Fusarium* sp. JZB3110090 + *D. macrodidyma* JZB3310008*;* **I** *Fusarium* sp. JZB3110089 + *D. macrodidyma* JZB3310008; **J** *Fusarium* sp. JZB3110102 + *D. macrodidyma* JZB3310008, **K** *Fusarium* sp. JZB3110103 + *D. macrodidyma* JZB3310008, **L** *Fusarium* sp. JZB3110180 + *D. macrodidyma* JZB3310008. The red arrow shows dark brown necrotic spots

To further explore the role of *Fusarium* in GTD occurrence, 104 *Fusarium* strains were isolated and identified from asymptomatic roots, rhizosphere soils, and bulk soils. The pathogenicity levels of five representative *Fusarium* strains were then evaluated. Grapevines inoculated with *Fusarium* isolates exhibited chlorotic leaf spots that gradually coalesced and turned orange, finally becoming necrotic (Additional file 2: Fig. S9B–F). All *Fusarium* isolates were able to cause dark brown necrosis on stems (Fig. 7B–F), in addition to root rot that blackened lateral roots. These symptoms were similar to those observed after inoculation with the GTD-associated fungus *D. macrodidyma* JZB3310008 (Fig. 7G).

To further investigate the relationship between *Fusarium* and GTD occurrence, grapevines were inoculated with *Fusarium* isolates and *D. macrodidyma* JZB3310008. Similar symptoms were subsequently observed on roots, stems, and leaves after inoculation with *Fusarium* isolates and *D. macrodidyma* (Fig. 7H–L, Additional file 2: Fig. S9H–L). Comparison of the disease severity caused by *Fusarium* spp. and *D. macrodidyma* JZB3310008, in addition to co-inoculations of the two, revealed that the disease symptoms caused by most *Fusarium* spp. were stronger than those caused by *D. macrodidyma* JZB3310008. In addition, the disease indices were higher in the co-inoculation treatment than under single inoculation with a *Fusarium* isolate or *D. macrodidyma* JZB3310008 (Additional file 1: Table S9). These results suggest that *Fusarium* spp. are the pathogens associated with GTDs, and that these pathogens can exacerbate disease severity when inoculated with other known GTD-associated pathogens.

## Discussion

### Factors affecting the composition of grapevine microbiota

In this study, fungal microbiomes associated with three soil–plant components (roots, including the rhizoplane and endophytes; rhizosphere soil; and bulk soil) of adjacent GTD-asymptomatic and symptomatic Chardonnay cultivar grapevines were collected in two consecutive years in China. Three factors, including the sampling year, different soil–plant compartments, and the presence/absence of GTD symptoms, were considered when comparing the fungal microbiome structures of belowground grapevine components. This study represents the first report to comparatively assess fungal communities among different soil–plant compartments of asymptomatic and symptomatic grapevines collected in different years using a high-throughput sequencing community-profiling approach. The results suggest that soil–plant compartments have significant effects on fungal communities, followed to a lesser extent by sampling year and symptomatology. GTDs are highly complex, and the pathogens associated with these diseases are usually present in latent states in plants without GTD symptoms. Furthermore, GTDs occurred unpredictably in different years [40]. Therefore, understanding the interannual differences of microbiomes, particularly the changes in GTD pathogen abundance, is helpful for further understanding the occurrence and mechanisms underlying GTDs.

Fungal communities are influenced by plant developmental stage [41]. Thus, samples were collected from the same vines and from the same growing stage over two consecutive years. Fungal communities and GTD-associated fungal populations significantly differed between years. These differences may possibly be due to changes in climate and environmental conditions between years, consistent with other studies demonstrating that microbiome composition is related to environmental factors such as climate and moisture levels [41–45]. The dominant GTD pathogens identified in this study included *I. liriodendra* in 2019 and *D. torresensis* in 2020. Most of the GTD-associated fungi detected in this study exhibited significantly different relative abundances between 2019 and 2020. Thus, the diversity of GTD-associated pathogens in the belowground components of grapevines is very high, and dominant populations can vary significantly year to year. Consequently, it cannot be definitively concluded that pathogens isolated from tissues near necrotic spots are the actual causative agents of necrosis.

The results of this study suggest that the soil–plant compartment is an important factor that shapes belowground microbiota composition, consistent with results of a recent study [46]. Most of the GTD pathogens identified in this study were prevalent in the roots and in the rhizosphere. These taxa may affect grapevines because the zones within roots and the rhizosphere are the main areas in which plant–microbe interactions occur. However, *Coprinellus* spp. associated with ESCA [47] were prevalent in bulk soils. Plant replacement is a widely used practice when GTDs appear. A small number of GTD pathogenic species were detected in belowground zones, and the enrichment of GTD-associated taxa in bulk soils indicated that tree replacement could still carry a high risk of disease because soils might be the primary source of inoculum [21].

### Correlation between the relative abundances of GTD pathogens in belowground grapevine compartments and symptomatology

To explore potential critical flora associated with GTDs, fungal community compositions associated with asymptomatic and symptomatic samples were compared when considering the same conditions. Significant differences were observed in the fungal community compositions of root and rhizosphere samples, but not in bulk soils, when comparing asymptomatic and symptomatic plants. Similar results were observed when comparing the fungal community compositions of asymptomatic and symptomatic bulk soils of an ESCA-infected vineyard [27]. The OTUs associated with GTDs detected in this study were assigned as 16 species and 17 genera. A previous study evaluating the wood fungal communities of a 17-year-old grapevine revealed a total of 10 fungi previously described as being responsible for GTDs [48]. In addition, 7–11 species and 11–14 genera associated with GTDs were detected in the cordon and trunk tissues of the cultivars ‘Agiorgitiko’, ‘Vidiano’, and ‘Xinomavro’ [11]. Although GTDs mainly occur in stems, greater numbers of pathogenic species were identified in belowground components in this study compared to aboveground components in other studies, suggests that soils may serve as GTD pathogen inoculum reservoirs. Nevertheless, comprehensive studies comparing the distribution of GTD-associated pathogens in aboveground and belowground components are needed to evaluate this hypothesis. Additionally, similar relative abundances of each GTD-associated pathogen detected in this study were observed between asymptomatic and symptomatic plants from the same soil–plant compartment collected in the same year. *Neonectria* and *Coprinellus* are pathogens that cause black foot disease and ESCA, respectively, and only their relative abundances significantly differed between asymptomatic and symptomatic rhizospheres collected in 2019. However, in contrast to expectations, their abundances were greater in asymptomatic samples than in symptomatic samples (Fig. 6, Additional file 1: Table S4). In addition, the total relative abundances of all GTD pathogens tested in the present study were higher in asymptomatic samples than symptomatic ones (Fig. 5, Additional file 1: Table S7). This result was consistent with previous studies showing that the relative abundances of GTD pathogens were significantly higher in asymptomatic spurs than in symptomatic ones [48]. However, other studies have reported that the abundances of GTD pathogens were positively correlated with GTDs in bulk soils [27] and wood in a cultivar/viticultural zone-dependent manner [11]. These observations suggest that the presence of GTD pathogens provides a possibility, but not the inevitability, for the occurrence of GTDs. Additional comprehensive studies are needed to confirm this assertion, because the compositions of GTD pathogens are affected by factors including climate [49], region, grape variety, nursery, rootstock [16, 50], environmental factors [42], grapevine developmental stages [41, 51]. Moreover, additional pathogens may be identified with further studies. Quantifying the overall fungal load would be useful for further investigations of the differences associated with GTD disease onset, as comparisons of the relative abundance between different samples may not reflect true differences.

### *Fusarium* spp. contribute to the development of GTDs

*Fusarium* spp*.* are important plant pathogens that can cause root rot and *Fusarium* wilt. They are saprophytic fungi that can survive in soils between crop cycles in infected plant debris, infecting the roots, the stems of the nodes, or the soil, leading to opportunistic plant wound infections [52, 53]. The results of this study indicate that *Fusarium* spp. are prevalent in grapevines, and that *Fusarium* spp. are part of the core grapevine microbiota across years and root–soil compartments, in addition to grapevine developmental stages and organs, as also suggested by previous research [41]. In addition, *Fusarium* spp. has been isolated from symptomatic and asymptomatic grapevine wood [54]. *Fusarium* spp. have been reported to potentially act as weak or latent pathogens in grapevines, as they are involved in grapevine decline, especially under favorable conditions [55, 56]. Because *Fusarium* spp. are common soil saprophytes and GTDs typically occur in the trunk regions, they are not typically associated with GTDs. Here, *Fusarium* spp. enrichment in symptomatic roots and rhizospheres were identified using microbiota analysis, and pathogenicity tests demonstrated that these species were associated with GTDs. The higher relative abundances of GTD pathogens and *Fusarium* spp. in 2020 samples were consistent with a higher GTD incidence rate in 2020 (data not shown). Similar to these results, higher relative abundances of *Fusarium* have been observed in the bulk soils [27] and wood tissues [48] of symptomatic grapevines than in asymptomatic grapevine samples, highlighting the potentially important roles of *Fusarium* spp. in GTD development.

Several studies have shown that the co-infection of pathogens associated with black foot disease alongside pathogens associated with Botryosphaeria dieback [57] or Petri disease [58] lead to increased disease severity. Similarly, the co-infection of pathogens causing black foot disease and *Fusarium* spp. in this study was also associated with increased GTD severity. Nevertheless, this study provides new insights into the mechanisms of belowground fungal communities affecting the development of GTDs from an ecological perspective. Moreover, the results of this study point to the need to control GTD-causing pathogens in soils, in addition to within trunks, as traditionally conducted.In addition of *Fusarium* spp,, several taxa were also enriched in symptomatic rhizosphere (Fig. 4B). Among them, Glomerelalles and *Plectosphaerella* which includes numerous plant pathogenic fungal species that were associated with plant root and collar rots of horticultural crops [59, 60]. This suggested that the enrichment of Glomerelalles and *Plectosphaerella* could also be related to GTD occurrence but further experiments are needed.

## Conclusions

In conclusion, a comprehensive comparison of fungal communities in the belowground components (roots, rhizospheres, and bulk soils) of asymptomatic and symptomatic grapevines was conducted here for the first time, with samples collected over two consecutive years from a vineyard with GTD presence. The fungal community diversity and composition differs according to the soil–plant compartments, sampling year and GTD symptomatology. Moreover, diverse GTD-associated fungi were detected in belowground components, suggesting that soils might act as the source of pathogen inocula. However, the relative abundances of GTD pathogens were not correlated (or were negatively correlated) with symptoms, suggesting that other yet-to-be identified factors could be involved in GTD development. Moreover, *Fusarium* spp. were enriched in symptomatic root and rhizosphere samples and were associated with GTDs after pathogenicity tests. This is the first report showing that *Fusarium* can exacerbate GTD severity, This study contributes to a better understanding of GTD development and highlights the important effects of *Fusarium* spp. in causing and exacerbating GTDs.

## Supplementary Information

**Additional file 1: Table S1.** The environmental temperature conditions during the seasons of 2019 and 2020. **Table S2.** The physical and chemical characteristics of the soil in the sampling vineyard. **Table S3.** The records of chemical agents and fertilizers in the sampling vineyard. **Table S4.** The reads numbers used to analyze in this study. **Table S5.** Alpha diversity index values for fungal communities in different soil–plant compartments. PE: rhizoPlane and Endophyte; R: rhizosphere; and B: bulk soils. Samples were collected from grapevine trunk disease (GTD) asymptomatic (As) and symptomatic (S) grapevines in 2019 (0.19) and 2020 (0.20). **Table S6.** Operational taxonomic units (OTUs) specific to asymptomatic or symptomatic samples. **Table S7.** The relative abundances of grapevine trunk disease (GTD)-associated fungi annotated at the species and genus levels detected with ITS amplicon sequencing data. **Table S8.** Grapevine trunk disease (GTD)-associated fungi exhibiting significant differences in relative abundance between comparison groups based on ITS amplicon sequencing. Statistical differences were evaluated with Wilcoxon tests. **Table S9.** Disease index values for grapevines inoculated with isolates in this study.

**Additional file 2: Fig. S1.** Typical grapevine trunk disease (GTD) symptoms in grapevines collected in this study. **A** The overall condition of symptomatic grapevines with yellow spots or tiger stripes on the leaves of multiple branches. **B** Foliar symptoms that initially appeared as chlorotic spots and subsequently coalesced and finally became necrotic. **C** Internal symptoms of the same vine with foliar symptoms showing dark brown necrosis. **Fig. S2.** Species accumulation curves for fungal communities based on ITS sequencing. **Fig. S3.** Boxplots illustrating differences in **A** Shannon, **B** Simpson, **C** Chao1, and **D** ACE diversity measures of fungal communities in soil–plant compartments in 2019 and 2020. Wilcoxon tests were used for statistical analyses. *0.01 < *p* < 0.05; **0.001 < *p* < 0.01; ***p < 0.001. **Fig. S4.** Venn diagram illustrating the overlap in the number of operational taxonomic units (OTUs) identified in fungal microbiota among sampling years in **A** roots, **B** rhizospheres, and **C** bulk soils. **D** and **E** show the overlap in OTUs among soil–plant compartment samples collected in 2019 and 2020, respectively. **Fig. S5.** The core microbiome of grapevine belowground soil–plant component communities. The flower plot **A** shows the core operational taxonomic units (OTUs) shared by all groups evaluated in this study. **B** and **C** show the relative abundances of the 10 most abundant fungal taxa belonging to the core microbiomes at the family and genus levels, respectively. The less abundant taxa are referred to as “others”. **Fig. S6.** Boxplots showing differences in **A** Shannon, **B** Simpson, **C** Chao1, and **D** ACE diversity values for fungal communities when comparing samples from asymptomatic and symptomatic grapevines. Wilcoxon tests were used for statistical analysis. *0.01 < *p* < 0.05; **0.001 < *p* < 0.01; ***p < 0.001. **Fig. S7.** Venn diagram showing the overlap of operational taxonomic units (OTUs) identified in fungal microbiota among asymptomatic and symptomatic roots (**A**), rhizospheres (**B**), and bulk soils (**C**). **D** The overlap of OTUs among asymptomatic and symptomatic samples when all asymptomatic and symptomatic samples were combined for analysis. **Fig. S8.** Ternary plot showing the relative abundances of grapevine trunk associated (GTD)-associated fungal genera in roots (PE), rhizospheres, and bulk soils. **A** Asymptomatic samples collected in 2019. **B** Symptomatic samples collected in 2019. **C** Asymptomatic samples collected in 2020. **D** Symptomatic samples collected in 2020. The size of the circles reflects the relative abundance. **Fig. S9.** Grapevine leaf symptoms after inoculation with *Fusarium* and *Dactylonectria macrodidyma* isolates. Inoculation with **A** control, **B** *Fusarium* sp. JZB3110090; **C** *Fusarium* sp. JZB3110089; **D** *Fusarium* sp. JZB3110102; **E** *Fusarium* sp. JZB3110103; **F** *Fusarium* sp. JZB3110180; **G** *D. macrodidyma* JZB3310008 associated with black foot disease; **H** *Fusarium* sp. JZB3110090 + *D. macrodidyma* JZB3310008*;* **I** *Fusarium* sp. JZB3110089 + *D. macrodidyma* JZB3310008; **J** *Fusarium* sp. JZB3110102 + *D. macrodidyma* JZB3310008, **K** *Fusarium* sp. JZB3110103 + *D. macrodidyma* JZB3310008, **L** *Fusarium* sp. JZB3110180 + *D. macrodidyma* JZB3310008. Interveinal discolorations were observed on leaves earlier and gradually coalesced to necrosis.

## Data Availability

The datasets generated during the current study are available from the corresponding author on reasonable request.

## Abbreviations

As: Asymptomatic
AsB.19: The bulk soil samples collected from asymptomatic grapevines in 2019
AsB.20: The bulk soil samples collected from asymptomatic grapevines in 2020
AsR.19: The rhizosphere samples collected from asymptomatic grapevines in 2019
AsR.20: The rhizosphere samples collected from asymptomatic grapevines in 2020
AsPE.19: The rhizoplane and endophyte samples collected from asymptomatic grapevines in 2019
AsPE.20: The rhizoplane and endophyte samples collected from asymptomatic grapevines in 2020
B: Bulk soils
ESCA: Esca complex disease
GTDs: Grapevine Trunk Diseases
LEfSe: Linear discriminant analysis effect size
OTU: Operational taxonomic units
PCoA: Principal coordinate analyses
PE: RhizoPlane and Endophyte
PERMANOVA: Permutational multivariate analyses of variance
R: Rhizosphere
S: Symptomatic
SB.19: The bulk soil samples collected from symptomatic grapevines in 2019
SB.20: The bulk soil samples collected from symptomatic grapevines in 2020
SR.19: The rhizosphere samples collected from symptomatic grapevines in 2019
SR.20: The rhizosphere samples collected from symptomatic grapevines in 2020
SPE.19: The rhizoplane and endophyte samples collected from symptomatic grapevines in 2019
SPE.20: The rhizoplane and endophyte samples collected from symptomatic grapevines in 2020
19: Samples collected in the year of 2019
20: Samples collected in the year of 2020
