# Supplementary material for: Belowground microbiota analysis indicates that Fusarium spp. exacerbate grapevine trunk disease
Source: Environ Microbiome. 2023 Apr 3;18:29. doi: 10.1186/s40793-023-00490-0 (PMC10071613; doi:10.1186/s40793-023-00490-0)
Supplement: Supplementary file 2 — Additional file 2: Fig. S1. Typical grapevine trunk disease (GTD) symptoms in grapevines collected in this study. A The overall condition of symptomatic grapevines with yellow spots or tiger stripes on the leaves of multiple branches. B Foliar symptoms that initially appeared as chlorotic spots and subsequently coalesced and finally became necrotic. C Internal symptoms of the same vine with foliar symptoms showing dark brown necrosis. Fig. S2. Species accumulation curves for fungal communities based on ITS sequencing. Fig. S3. Boxplots illustrating differences in A Shannon, B Simpson, C Chao1, and D ACE diversity measures of fungal communities in soil–plant compartments in 2019 and 2020. Wilcoxon tests were used for statistical analyses. *0.01 < p < 0.05; **0.001 < p < 0.01; ***p < 0.001. Fig. S4. Venn diagram illustrating the overlap in the number of operational taxonomic units (OTUs) identified in fungal microbiota among sampling years in A roots, B rhizospheres, and C bulk soils. D and E show the overlap in OTUs among soil–plant compartment samples collected in 2019 and 2020, respectively. Fig. S5. The core microbiome of grapevine belowground soil–plant component communities. The flower plot A shows the core operational taxonomic units (OTUs) shared by all groups evaluated in this study. B and C show the relative abundances of the 10 most abundant fungal taxa belonging to the core microbiomes at the family and genus levels, respectively. The less abundant taxa are referred to as “others”. Fig. S6. Boxplots showing differences in A Shannon, B Simpson, C Chao1, and D ACE diversity values for fungal communities when comparing samples from asymptomatic and symptomatic grapevines. Wilcoxon tests were used for statistical analysis. *0.01 < p < 0.05; **0.001 < p < 0.01; ***p < 0.001. Fig. S7. Venn diagram showing the overlap of operational taxonomic units (OTUs) identified in fungal microbiota among asymptomatic and symptomatic roots (A), rhizospheres (B), and bulk soil [file 40793_2023_490_MOESM2_ESM.pptx]

## Slide 1
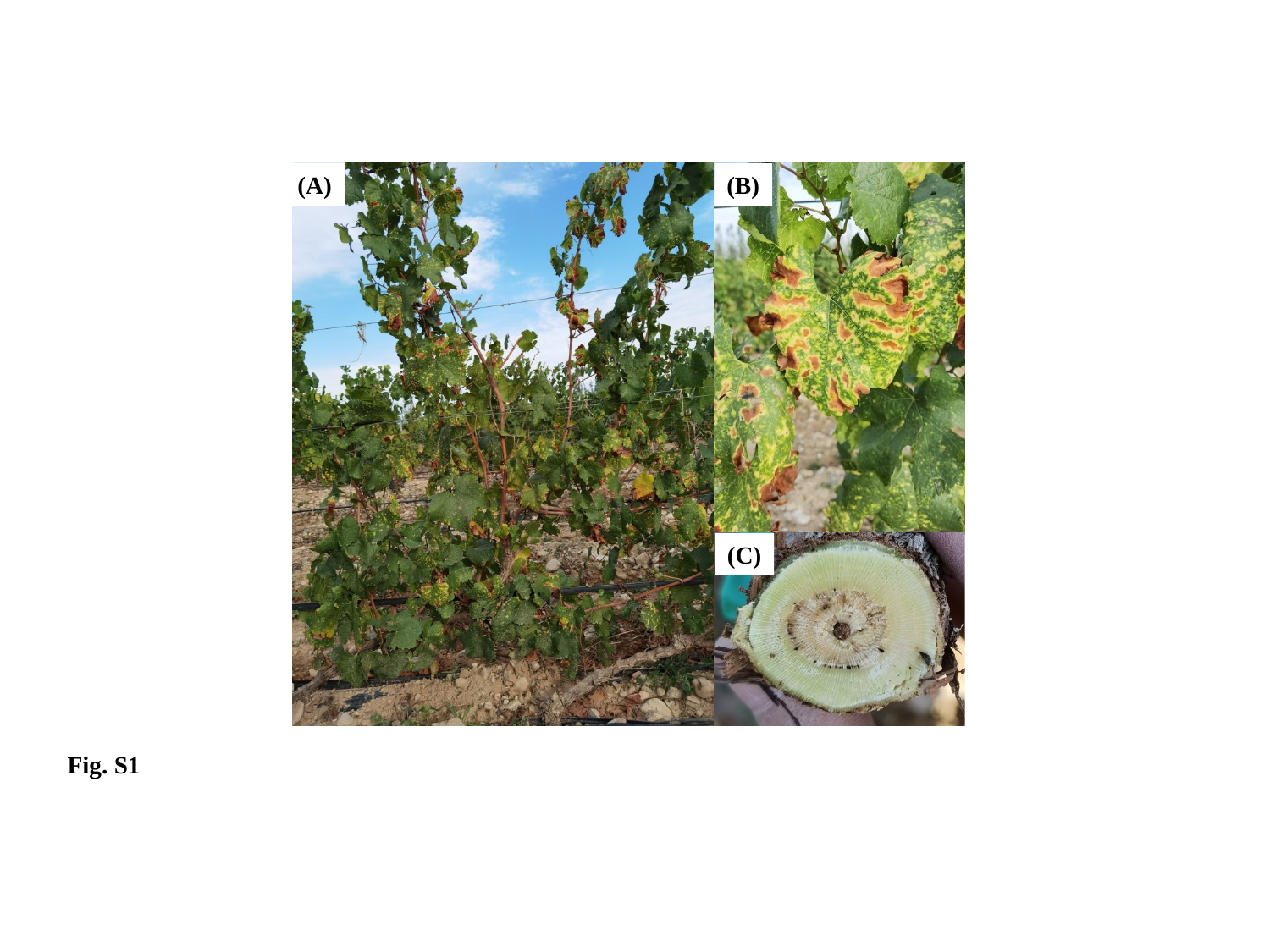

(A)
(B)
(C)
Fig. S1

## Slide 2
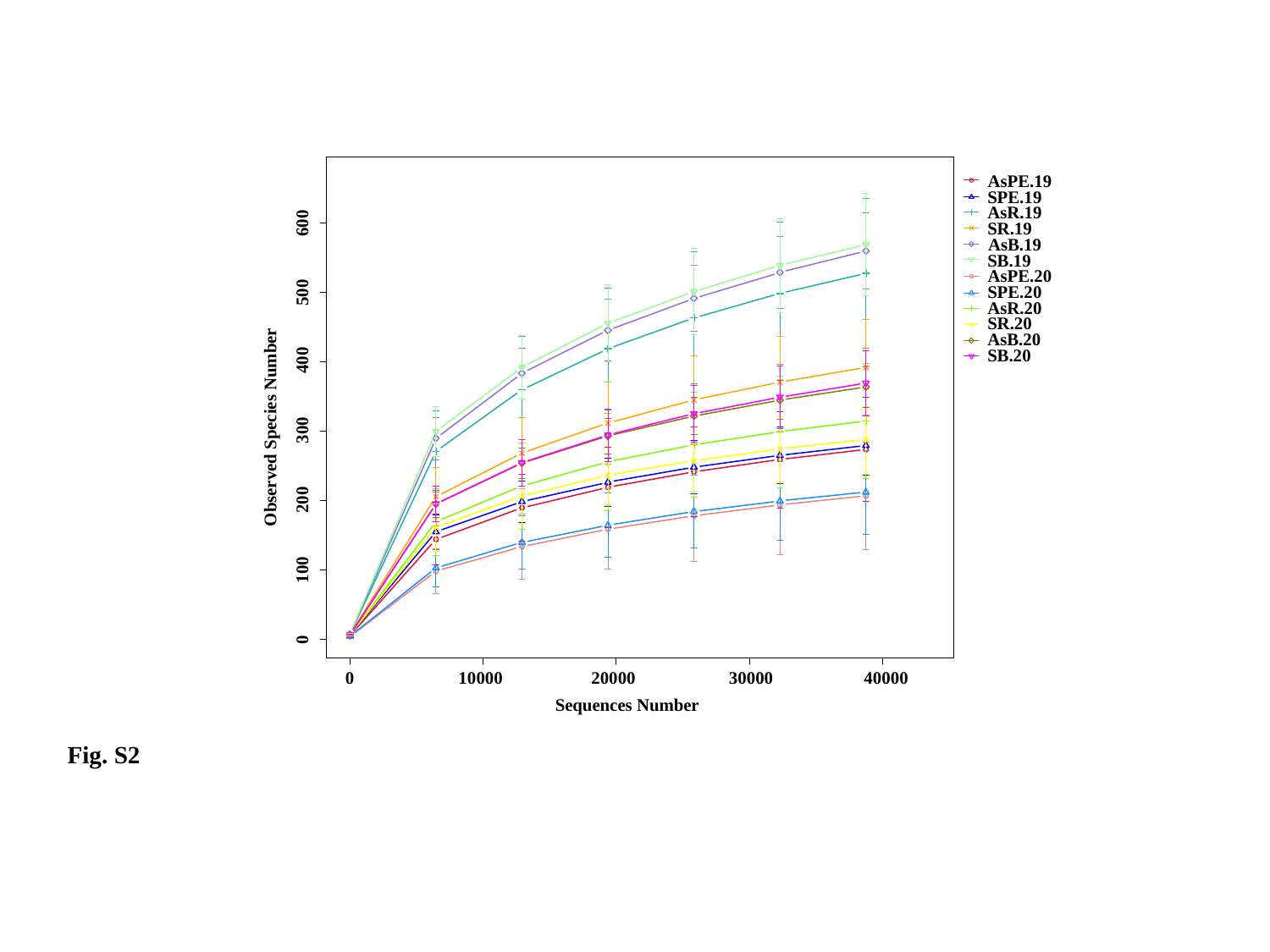

AsPE.19
SPE.19
AsR.19
SR.19
AsB.19
SB.19
AsPE.20
SPE.20
AsR.20
SR.20
AsB.20
SB.20
600
500
400
Observed Species Number
300
200
100
0
0
10000
20000
30000
40000
Sequences Number
Fig. S2

## Slide 3
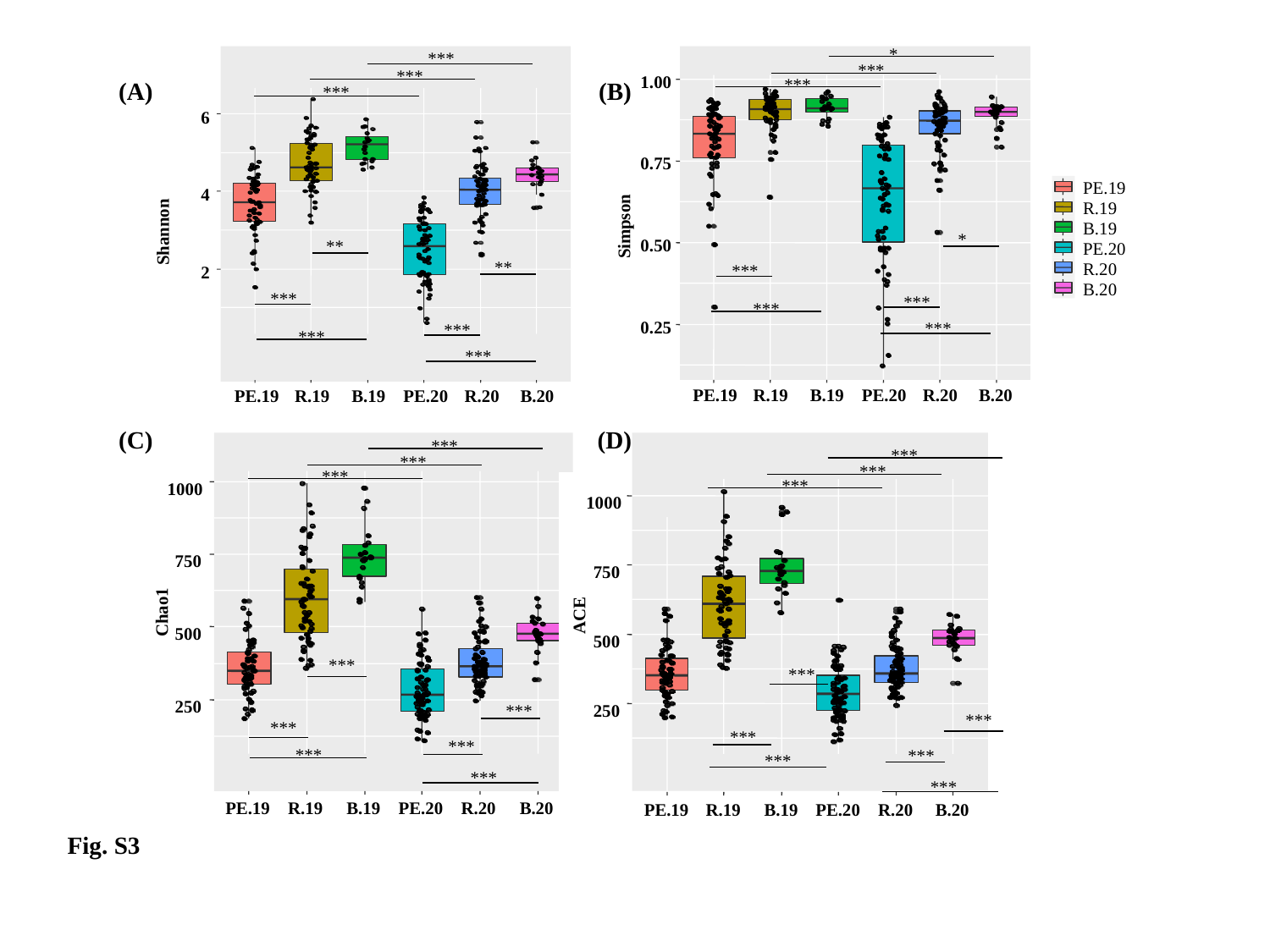

*
***
***
*
***
***
***
***
***
***
(A)
***
6
4
Shannon
**
**
2
***
***
***
***
PE.19
R.19
B.19
PE.20
R.20
B.20
1.00
0.75
PE.19
R.19
B.19
Simpson
0.50
PE.20
R.20
B.20
0.25
PE.19
R.19
B.19
PE.20
R.20
B.20
(B)
(C)
***
***
***
***
***
***
***
***
***
1000
750
Chao1
500
250
PE.19
R.19
B.19
PE.20
R.20
B.20
(D)
1000
750
ACE
500
250
R.20
B.20
PE.19
R.19
B.19
PE.20
***
***
***
***
***
***
***
***
***
Fig. S3

## Slide 4
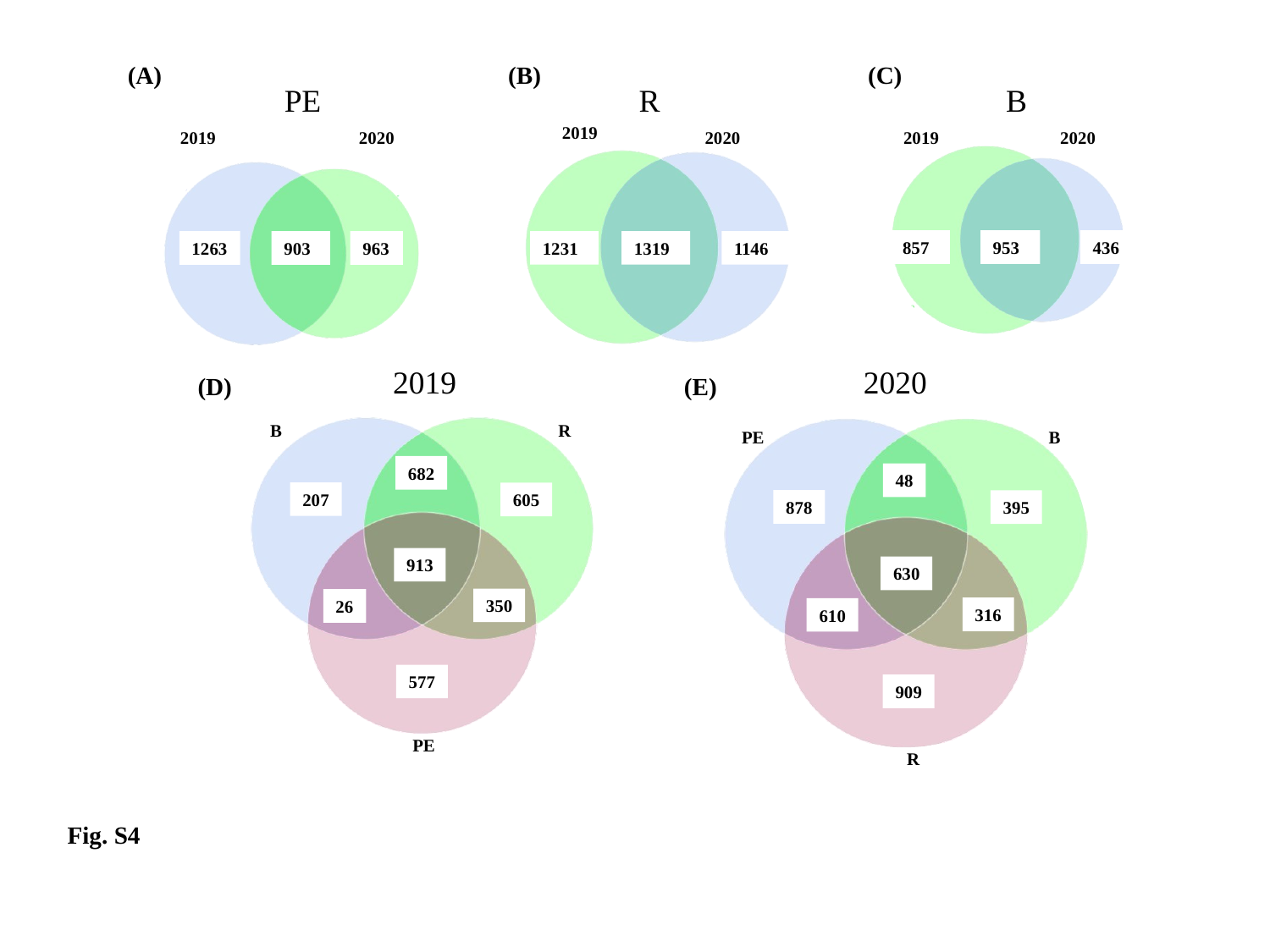

(A)
(B)
(C)
PE
R
B
2019
2019
2020
2020
2019
2020
857
953
436
1231
1319
1146
1263
903
963
2019
2020
(D)
(E)
PE
B
48
878
395
630
316
610
909
R
B
R
PE
682
207
605
913
350
26
577
Fig. S4

## Slide 5
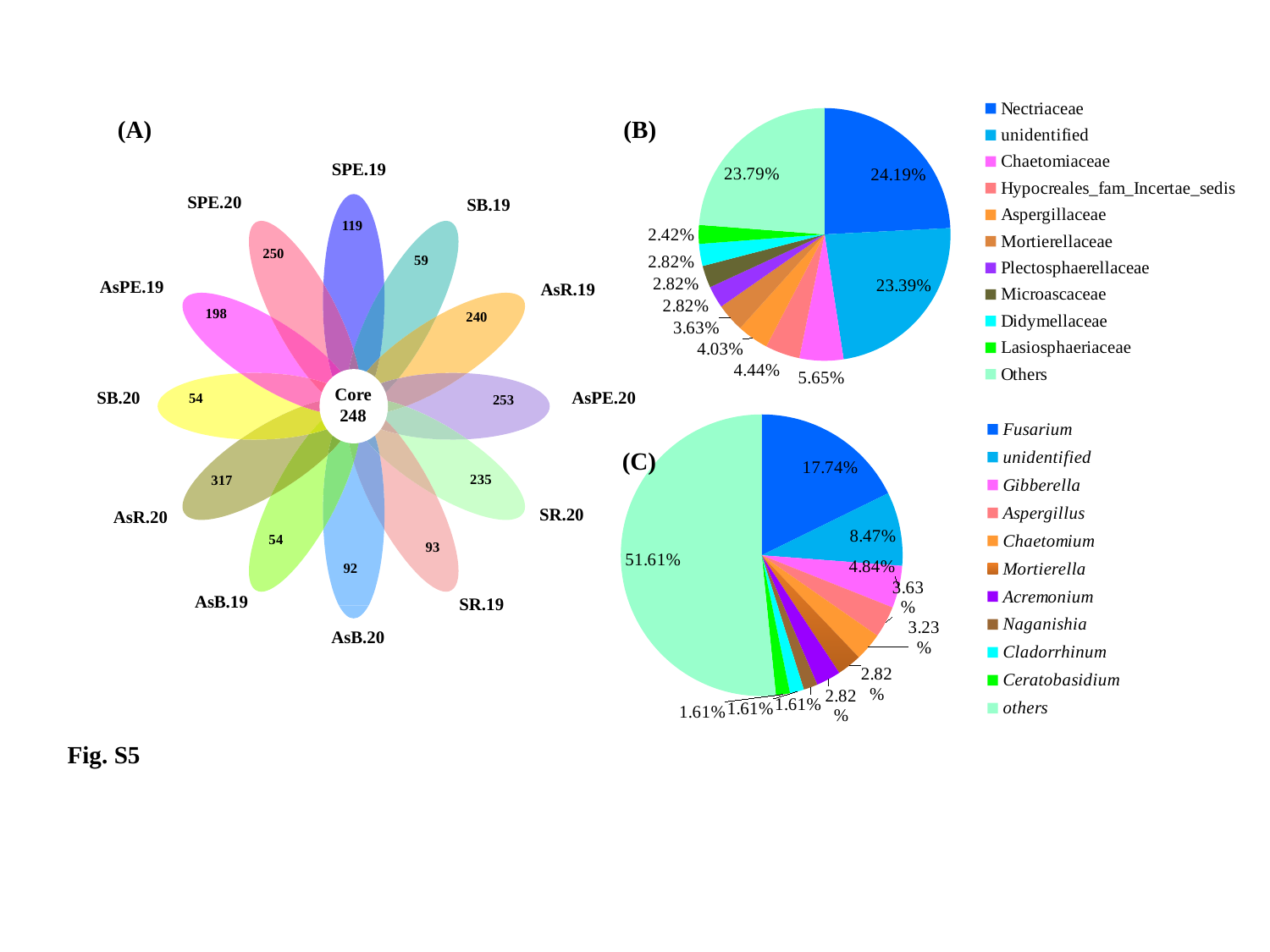

### Chart
| Category | |
|---|---|
| Nectriaceae | 0.241935483870968 |
| unidentified | 0.233870967741935 |
| Chaetomiaceae | 0.0564516129032258 |
| Hypocreales_fam_Incertae_sedis | 0.0443548387096774 |
| Aspergillaceae | 0.0403225806451613 |
| Mortierellaceae | 0.0362903225806452 |
| Plectosphaerellaceae | 0.0282258064516129 |
| Microascaceae | 0.0282258064516129 |
| Didymellaceae | 0.0282258064516129 |
| Lasiosphaeriaceae | 0.0241935483870968 |
| Others | 0.237903225806452 |(A)
(B)
SPE.19
SPE.20
SB.19
119
250
59
AsPE.19
AsR.19
198
240
Core
248
AsPE.20
SB.20
54
253
235
317
SR.20
AsR.20
54
93
92
AsB.19
SR.19
AsB.20
### Chart
| Category | |
|---|---|
| Fusarium | 0.17741935483871 |
| unidentified | 0.0846774193548387 |
| Gibberella | 0.0483870967741936 |
| Aspergillus | 0.0362903225806452 |
| Chaetomium | 0.032258064516129 |
| Mortierella | 0.0282258064516129 |
| Acremonium | 0.0282258064516129 |
| Naganishia | 0.0161290322580645 |
| Cladorrhinum | 0.0161290322580645 |
| Ceratobasidium | 0.0161290322580645 |
| others | 0.516129032258065 |(C)
Fig. S5

## Slide 6
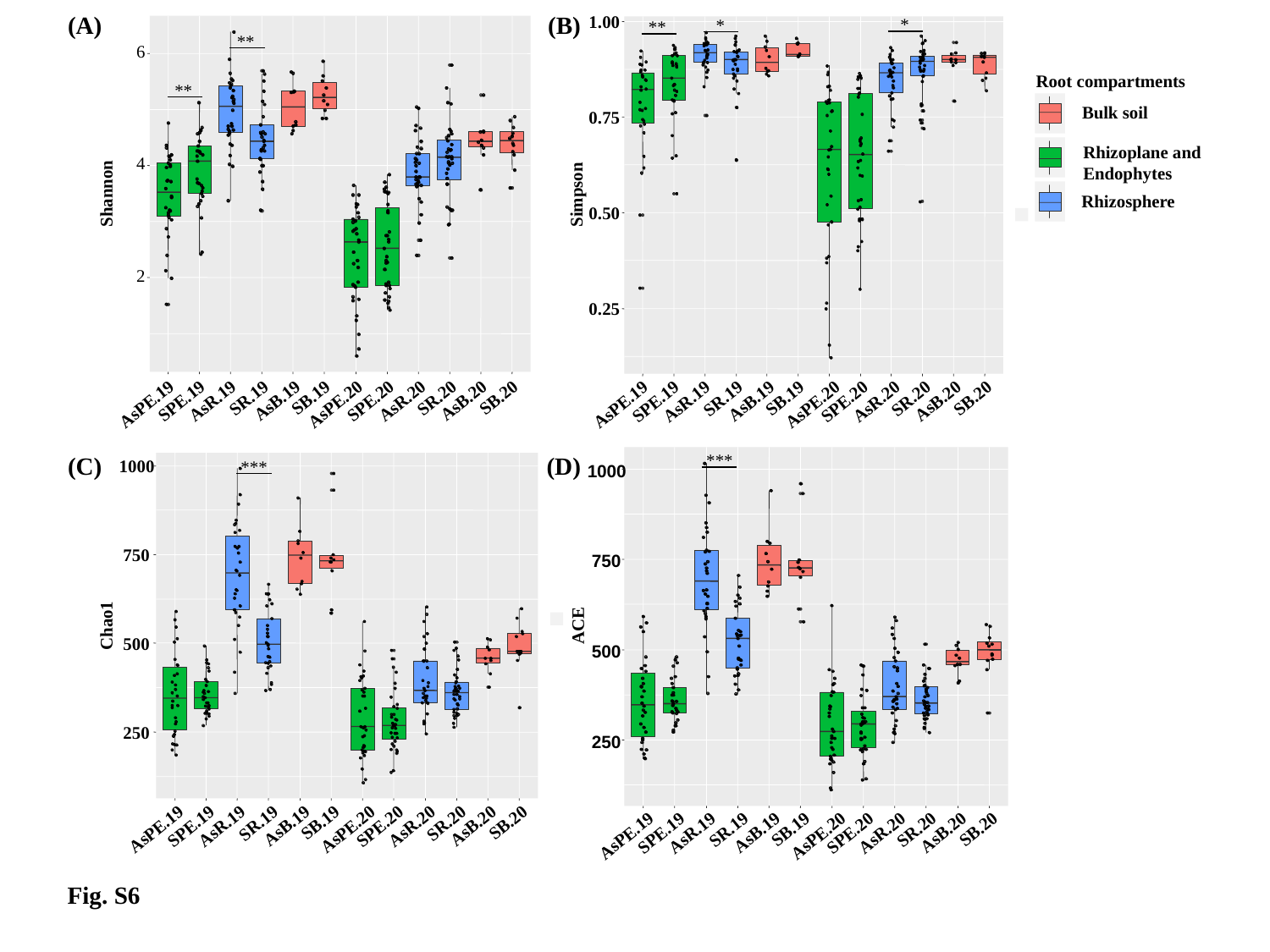

(A)
(B)
*
*
**
1.00
0.75
Simpson
0.50
0.25
SB.19
SB.20
SR.19
SR.20
AsB.19
AsB.20
SPE.19
AsR.19
SPE.20
AsR.20
AsPE.19
AsPE.20
**
6
Root compartments
**
Bulk soil
4
Shannon
Rhizosphere
2
SB.19
SB.20
SR.19
SR.20
AsB.19
AsB.20
SPE.19
AsR.19
SPE.20
AsR.20
AsPE.19
AsPE.20
***
1000
750
ACE
500
250
SB.19
SB.20
SR.19
SR.20
AsB.19
AsB.20
SPE.19
AsR.19
SPE.20
AsR.20
AsPE.19
AsPE.20
(C)
(D)
1000
750
Chao1
500
250
SB.19
SB.20
SR.19
SR.20
AsB.19
AsB.20
SPE.19
AsR.19
SPE.20
AsR.20
AsPE.19
AsPE.20
***
Rhizoplane and Endophytes
Fig. S6

## Slide 7
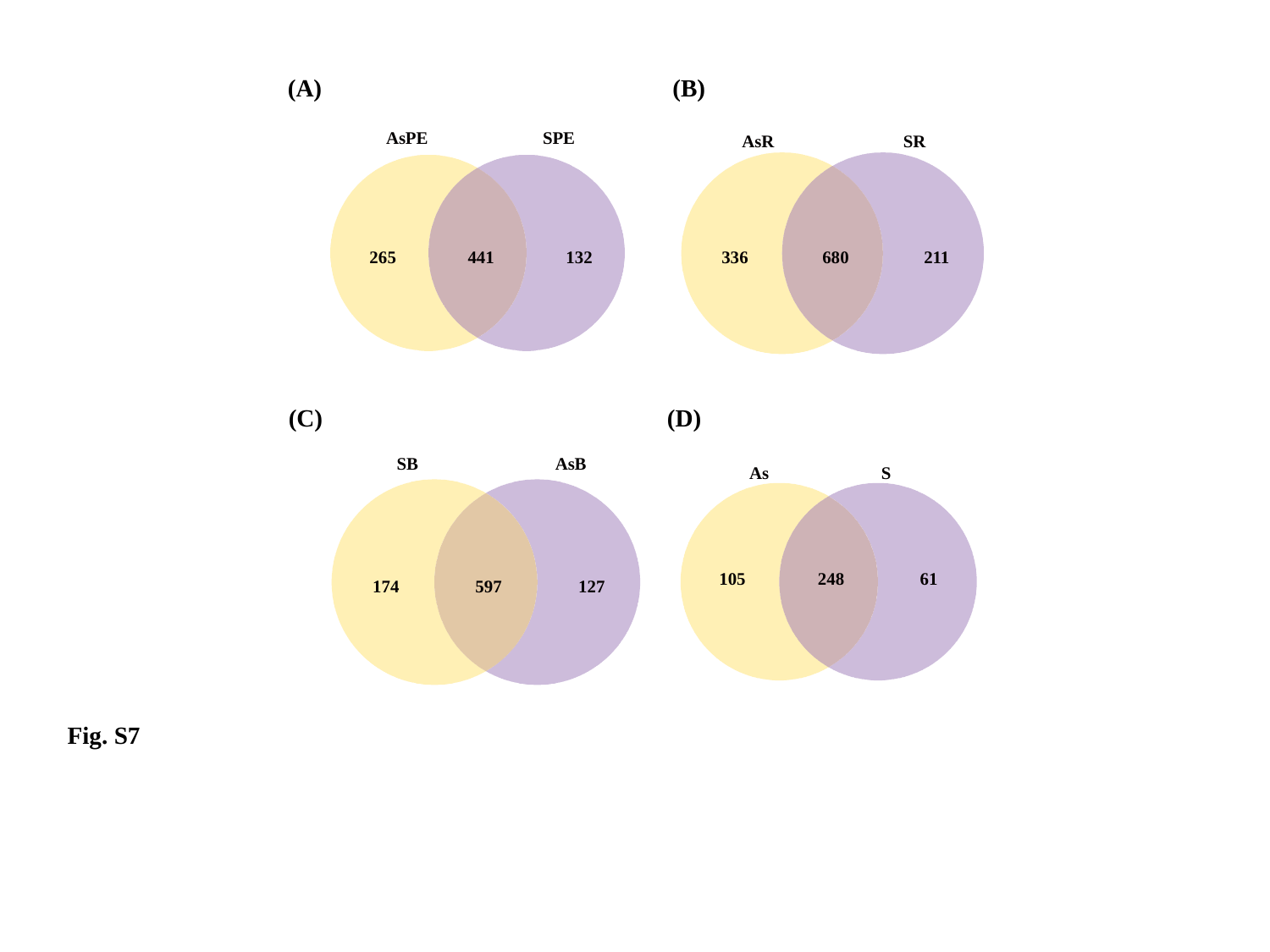

(A)
(B)
AsR
SR
336
680
211
AsPE
SPE
265
441
132
(C)
(D)
SB
AsB
174
597
127
As
S
105
248
61
Fig. S7

## Slide 8
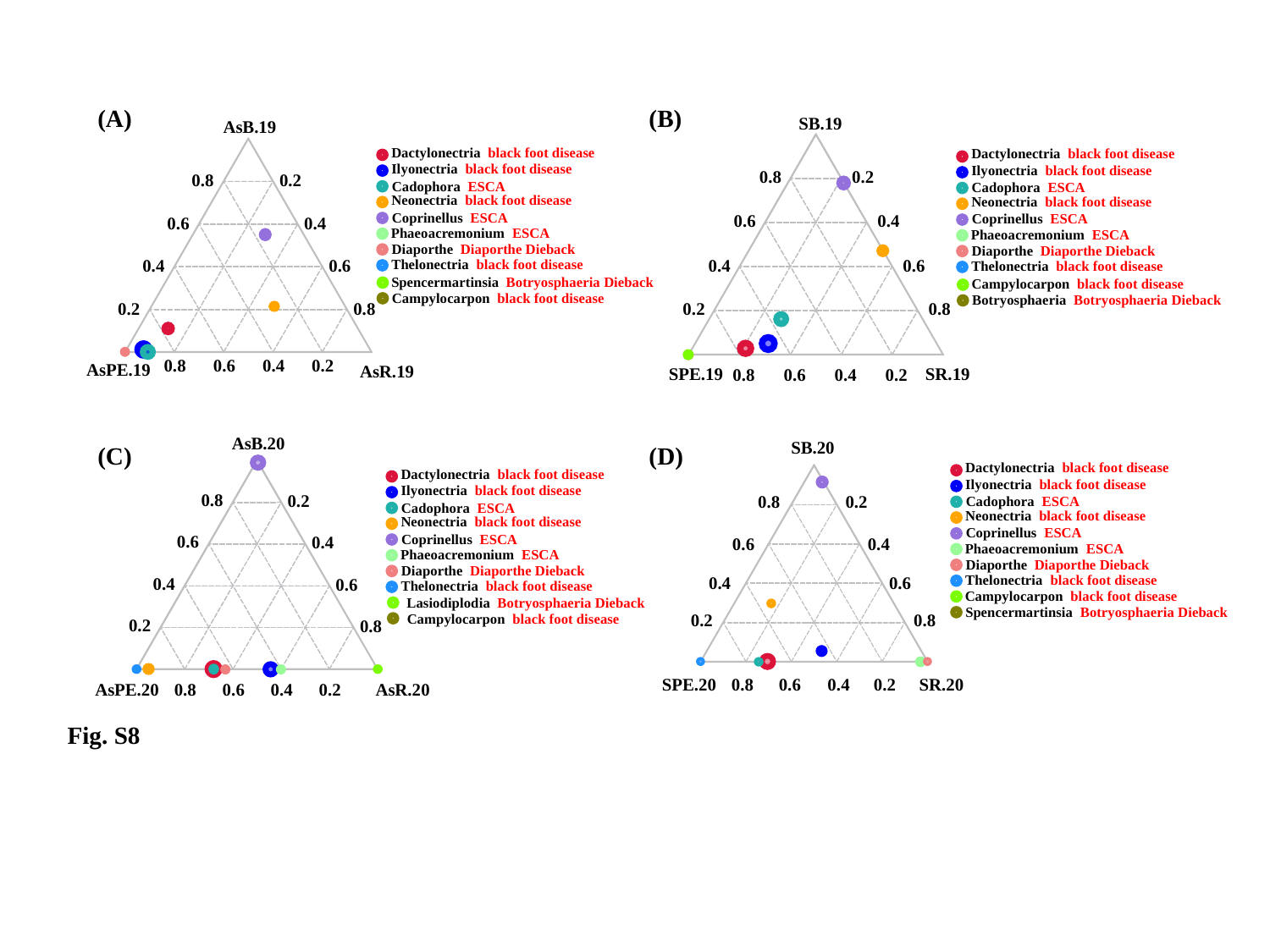

(A)
(B)
SB.19
Dactylonectria black foot disease
Ilyonectria black foot disease
Cadophora ESCA
Neonectria black foot disease
Coprinellus ESCA
Phaeoacremonium ESCA
Diaporthe Diaporthe Dieback
Thelonectria black foot disease
Campylocarpon black foot disease
Botryosphaeria Botryosphaeria Dieback
0.8
0.2
0.6
0.4
0.4
0.6
0.2
0.8
SPE.19
SR.19
0.8
0.6
0.4
0.2
AsB.19
Dactylonectria black foot disease
Ilyonectria black foot disease
Cadophora ESCA
Neonectria black foot disease
Coprinellus ESCA
Phaeoacremonium ESCA
Diaporthe Diaporthe Dieback
Thelonectria black foot disease
Spencermartinsia Botryosphaeria Dieback
Campylocarpon black foot disease
0.8
0.2
0.6
0.4
0.4
0.6
0.2
0.8
0.8
0.6
0.4
0.2
AsR.19
AsPE.19
AsB.20
Dactylonectria black foot disease
Ilyonectria black foot disease
Cadophora ESCA
Neonectria black foot disease
Coprinellus ESCA
Phaeoacremonium ESCA
Diaporthe Diaporthe Dieback
Thelonectria black foot disease
Lasiodiplodia Botryosphaeria Dieback
Campylocarpon black foot disease
0.8
0.2
0.6
0.4
0.4
0.6
0.2
0.8
AsPE.20
0.8
0.6
0.4
0.2
AsR.20
SB.20
Dactylonectria black foot disease
Ilyonectria black foot disease
Cadophora ESCA
Neonectria black foot disease
Coprinellus ESCA
Phaeoacremonium ESCA
Diaporthe Diaporthe Dieback
Thelonectria black foot disease
Campylocarpon black foot disease
Spencermartinsia Botryosphaeria Dieback
0.8
0.2
0.6
0.4
0.4
0.6
0.2
0.8
SPE.20
0.8
0.6
0.4
0.2
SR.20
(C)
(D)
Fig. S8

## Slide 9
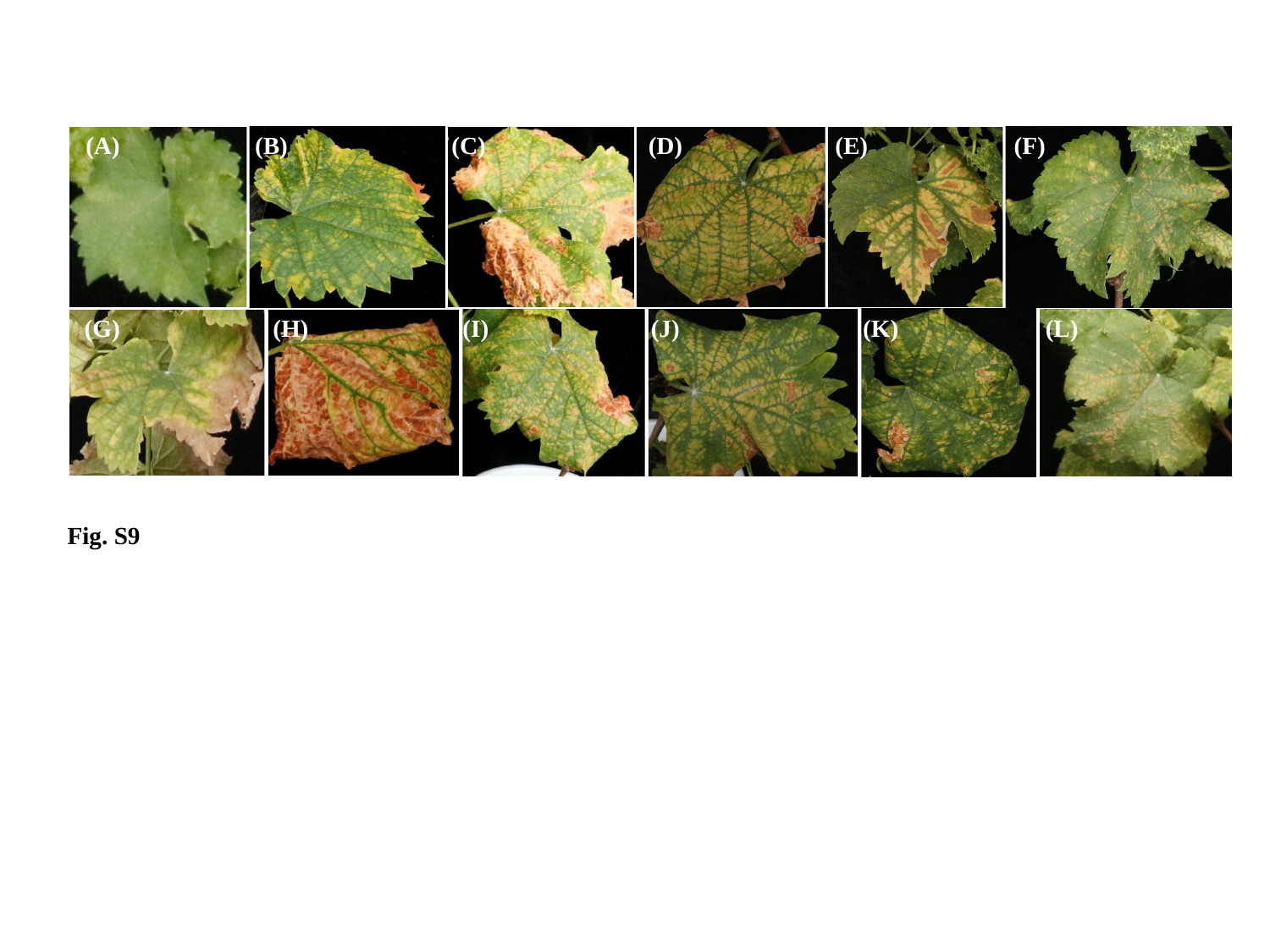

(A)
(B)
(C)
(D)
(E)
(F)
(G)
(H)
(I)
(J)
(K)
(L)
Fig. S9
